# The periplasmic domains of *Vibriocholerae* ToxR and ToxS are forming a strong heterodimeric complex independent on the redox state of ToxR cysteines

**DOI:** 10.1111/mmi.14673

**Published:** 2021-01-25

**Authors:** Nina Gubensäk, Gabriel E. Wagner, Evelyne Schrank, Fabio S. Falsone, Tamara Margot Ismael Berger, Tea Pavkov‐Keller, Joachim Reidl, Klaus Zangger

**Affiliations:** ^1^ Institute of Chemistry/Organic and Bioorganic Chemistry University of Graz Graz Austria; ^2^ Institute of Molecular Biosciences University of Graz Graz Austria; ^3^ Diagnostic and Research Institute of Hygiene, Microbiology and Environmental Medicine Medical University of Graz Graz Austria; ^4^ KAGes Healthcare Graz Austria; ^5^ BioTechMed‐Graz Graz Austria; ^6^ Field of Excellence BioHealth University of Graz Graz Austria

**Keywords:** ToxR, ToxS ‐ NMR, *Vibrio* *cholerae*

## Abstract

The transmembrane protein ToxR plays a key role in the virulence expression system of *Vibrio cholerae*. The activity of ToxR is dependent on its periplasmic sensor domain (ToxRp) and on the inner membrane protein ToxS. Herein, we present the Nuclear Magnetic Resonance NMR solution structure of the sensory ToxRp containing an intramolecular disulfide bond. The presented structural and dynamic experiments with reduced and oxidized ToxRp propose an explanation for the increased proteolytic sensitivity of reduced ToxR. Additionally, for the first time, we could identify the formation of a strong heterodimer complex between the periplasmic domains of ToxR and ToxS in solution. NMR interaction studies reveal that binding of ToxS is not dependent on the redox state of ToxR cysteines, and formed complexes are structurally similar. By monitoring the proteolytic cleavage of ToxRp with NMR, we additionally provide a direct evidence of ToxS protective function. Taken together our results suggest that ToxR activity is regulated by its stability which is, on the one hand, dependent on the redox states of its cysteines, influencing the stability of its fold, and on the other hand, on its interaction with ToxS, which binds independent on the cysteines and acts as a protection against proteases.

## Graphical abstract

This paper presents the first evidence of a heterodimer formation between the periplasmic domains of ToxR and ToxS, two main regulators of the cholera causative *Vibrio cholerae*. The interaction establishes a protection of ToxR against proteolysis. The atomic resolution structure of ToxRp shows an αβ‐fold followed by a long unstructured C‐terminal stretch which plays a significant role in the stability of ToxRp.

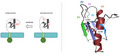

## 1 INTRODUCTION

The fatal diarrheal disease cholera is caused by the ingestion of the Gram‐negative bacterium *Vibrio cholerae*. Overall it accounts for 1.3–4.0 million infections and 21 000–143 000 deaths annually in 51 endemic countries (Ali et al., 2015).

The bacterium persists in aquatic habitats predominantly in a dormant state, which is described as viable but not culturable (Almagro‐Moreno et al., 2015a; Bari et al., 2013). When entering the human host, the bacterium rapidly switches to the active virulent state thereby enabling the survival and colonization of the bacterium in the human small intestine tract (Almagro‐Moreno et al., 2015b; Peterson and Mekalanos, 1988). The regulatory cascade, called ToxR‐ regulon (Matson et al., 2007) leads to the production of the main virulence factors namely the toxin co‐regulated pilus (TCP), and the cholera toxin (CT). While TCP is absolutely vital for the colonization in the small intestine (Taylor et al., 1987), it is the CT that triggers the diarrheal symptoms of the cholera disease (Sánchez and Holmgren, 2008; Taylor et al., 1987). Under such virulence inducing conditions, the conserved *toxRS* operon is constitutively expressed (Kanjilal et al., 2010), thereby regulating numerous genes (Bina et al., 2003; Champion et al., 1997; Lee et al., 2000; Skorupski and Taylor, 1997; Wang et al., 2002; Welch and Bartlett, 1998). ToxRS co‐activates, together with their homolog regulators TcpPH, the transcription of the main virulence regulator ToxT (Bina et al., 2018; Childers and Klose, 2007; Häse and Mekalanos, 1998; Higgins and DiRita, 1994; Krukonis et al., 2000). In contrast to TcpPH, which is encoded on the *Vibrio* Pathogenicity Island (VPI) (Jermyn and Boyd, 2002), ToxRS is also present in nonpathogenic isolates of *V. cholerae*, suggesting their involvement also in non‐virulent associated processes such as adaption to environmental stress conditions (Almagro‐Moreno et al., 2015a).

ToxR is built up of three domains, encompassing the periplasmic, membrane, and cytoplasmic compartments (Crawford et al., 2003; Miller et al., 1987; Osorio and Klose, 2000). Its activation is triggered by the sensing of environmental substances, like certain amino acids (Mey et al., 2012) or bile acids (Midgett et al., 2017), through its periplasmic domain. Bile acids are present in the small upper intestine tract and bind directly to the ToxR periplasmic sensory domain (Midgett et al., 2017; Midgett et al., 2020) thereby inducing a switch in the outer membrane porin expression from OmpT to OmpU (Miller and Mekalanos, 1988; Provenzano and Klose, 2000). The production of OmpU enables the bacterium to survive in the small intestine since it provides a more efficient exclusion of bile acids due to its narrow and negatively charged pore (Wibbenmeyer et al., 2002).

The cytoplasmic DNA binding domain of ToxR forms a winged helix‐turn‐helix motif (Martínez‐Hackert and Stock, 1997) which binds to the so called “tox‐boxes” as proposed dimers (Crawford et al., 1998; Goss et al., 2013; Krukonis and DiRita, 2003; Pfau and Taylor, 1996). Apparent dimerization of ToxR, as observed by overexpressed ToxR molecules, is achieved via the formation of intermolecular disulfide bonds between C236 and C293, located in the periplasmic domain of ToxR (Fengler et al., 2012; Lembke et al., 2020; Ottemann and Mekalanos, 1996). The formation of activity essential ToxR homodimers, containing intermolecular disulfide bonds, was shown to be dependent on periplasmic oxidoreductases DsbA/DsbC (Fengler et al., 2012; Lembke et al., 2020). Unexpectedly, the monomeric form of ToxR, containing the intra‐chain disulfide bridge, was found to be the predominant form in vivo and to be essential for the porin but not for *toxT* regulation (Fengler et al., 2012; Midgett et al., 2020).

The activity of ToxR depends on its effector ToxS (Almagro‐Moreno et al., 2015c; Miller et al., 1989), consisting of a periplasmic domain anchored in the inner membrane. Mutations of *toxS* negatively influence ToxR‐ToxR interactions in a homodimer and the regulation of ToxR dependent genes, including virulence factors (Lembke et al., 2020; Lembke et al., 2018). Deletions of *toxS* furthermore reveal an increased proteolysis of ToxR, which induces an entry of the bacterium into the dormant non‐virulent state of the bacterium (Almagro‐Moreno et al., 2015a; Almagro‐Moreno et al., 2015c; Pennetzdorfer et al., 2019). *In vitro* experiments showed a physical interaction between the periplasmic domains of both proteins (Midgett et al., 2017). However, the composition as well as the binding strength of the complex formation are not clarified. Since the activity of ToxR depends on its dimerization state and its interaction with ToxS, the question arises how binding of ToxS influences the oligomerization state of ToxR.

Interestingly, the reduced form of ToxR is the protease sensitive form as shown in *V. cholerae* cultures incubated with reducing agent DTT (Fengler et al., 2012; Lembke et al., 2018). Proteolysis of ToxR is mainly targeted by periplasmic proteases DegS and DegP (Lembke et al., 2018; Pennetzdorfer et al., 2019). In which way stabilization by ToxS depends on the redox state of ToxR is not completely understood yet.

**FIGURE 1:**
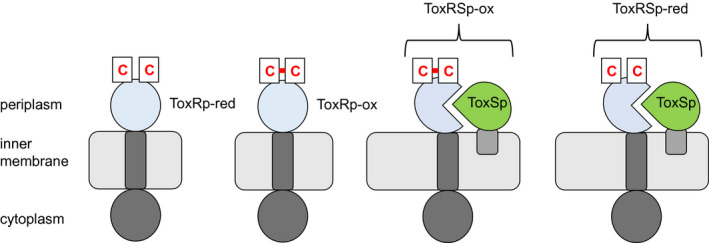
Schematic presentation of ToxR domains and its interaction with ToxS, both located at the inner membrane of *V. cholerae*. The two cysteines of the periplasmic domain of ToxR (ToxRp) can form an intramolecular disulphide bridge referred to as ToxRp‐ox, the reduced state is named ToxRp‐red. The periplasmic domain of ToxS (ToxSp) can form heterodimers with ToxRp‐ox (ToxRSp‐ox) and ToxRp‐red (ToxRSp‐red)

The versatile functions of ToxR propose separate control mechanisms, in order to regulate the activity of ToxR as direct activator, co‐activator, or repressor. Since the periplasmic sensory domain seems to be crucial in the initiation of ToxR activity, we concentrated on solving the structure of this highly interesting domain. Herein, we present the Nuclear Magnetic Resonance (NMR) solution structure of the periplasmic sensory domain of *V. cholerae* ToxR forming an intramolecular disulfide bond (ToxRp‐ox) (Figure 1). Furthermore, this study provides an explanation for the increased proteolytic sensitivity of the reduced form of ToxRp (ToxRp‐red) that was shown in *V. cholerae* (Lembke et al., 2020; Lembke et al., 2018). In addition, the obtained results offer new insights into the binding event of the virulence essential interaction of ToxR and ToxS. For the first time, we could identify the formation of a strong heterodimer between the periplasmic domains of ToxR (ToxRp) and ToxS (ToxSp) independent on the redox state of ToxRp. Our results reveal that ToxRp binds ToxSp in a 1:1 fashion with a dissociation constant of 11.6 nM. Additionally, by monitoring the proteolytic cleavage of ToxRp‐ox with NMR we provide a direct evidence of ToxS protective function.

## 2 RESULTS

### 2.1 NMR solution structure and dynamics of *V. cholerae* ToxRp‐ox

**FIGURE 2:**
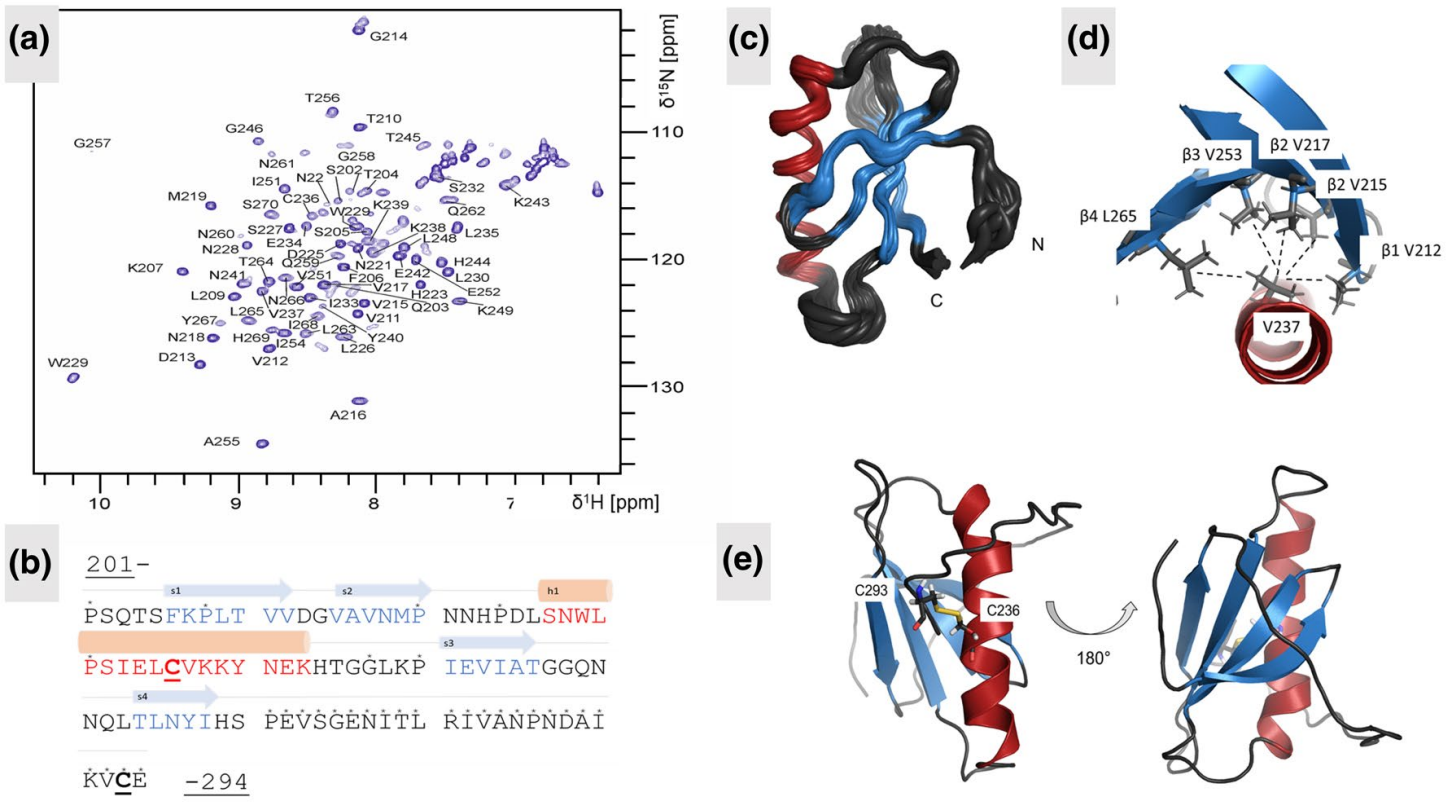
The NMR solution structure of ToxRp‐ox. (a): NMR backbone HN assignment of 2D ^15^N^‐^^1^H HSQC of ToxRp‐ox. (b): Amino acid sequence of the periplasmic domain of *V. cholerae* ToxR. Residues in red belong to alpha‐helical parts, residues in blue to beta strands. Residues marked with an asterisk are not visible in the ^15^N‐HSQC. The two cysteines (C236, C293), underlined in the sequence, are forming an intramolecular disulphide bond in the absence of reducing agents. (c): Bundle of 20 calculated NMR structures of ToxRp‐ox (excluding flexible N‐and C‐terminal residues). The average backbone RMSD to mean is 0.55 ± 0.14Å. The average heavy atom RMSD to mean is determined as 0.95 ± 0.17Å (d): Presentation of hydrophobic interactions of V237 located in the helix of ToxRp‐ox, close to the activity dependant C236 residue. V237 forms apolar contacts to all four beta strands and is therefore most probably essential for a correct fold of the protein. (e): Cartoon representation of ToxRp‐ox including the C‐terminal flexible region. The disulphide bond is formed between C236, in the middle of the helix, and C293 close to the C‐terminus. The disulphide bridge confines the movement of the unstructured C‐terminus. Due intermediate exchange on the NMR time scale the C‐terminal stretch is not visible in the ^15^N‐HSQC spectrum

ToxRp‐ox was excised from the chemically synthesized, *E. coli* codon optimized, full‐length ToxR gene and expressed with an N‐terminal 6x His tag, recombinantly in *E. coli* BL21 DE3 cells using ^15^N and ^13^C‐labeled minimal medium. After purification by His‐trap and size exclusion chromatography, the protein was investigated at a concentration of 0.5 mM in 50 mM NaPi buffer pH 6.5 100 mM NaCl in 90% H_2_O/10% D_2_O. The purity of a freshly made sample of ToxRp‐ox was established by SDS page. The 2D ^15^N‐^1^H HSQC spectrum (Figure 2a) shows mainly well‐dispersed signals, which indicates that the protein is mostly well‐folded.

The number of NH‐resonances was found to be lower than the number of amino acids, pointing to the presence of flexibility on the intermediate NMR time scale (i.e., molecular motions in the time range of milliseconds, which leads to extensive broadening and thereby disappearing NMR signals). Sequential backbone and side chain assignments localized the well‐dispersed signals to residues 201–270. The signals of the C‐terminal 24 residues were missing in the spectra (Figure 2b).

The solution structure of the monomeric oxidized sensory periplasmic domain of *V. cholerae* namely ToxRp‐ox was determined using NOEs and dihedral angles predicted based on chemical shifts by TALOS+ (Shen et al., 2009). It forms an αβ‐fold in solution, consisting of an alpha helix stacked against a four stranded beta sheet (Figure 2c,e). The beta strands form a four stranded beta sheet comprised by two hairpin motifs. Residues W229‐K243 are forming an α‐helix, the residues S227 and N228 are part of a beta turn type I and resemble an alpha‐helical like formation. There are two cysteines (C236 and C293) in the periplasmic domain of ToxR. C236 is in the middle of the helix of ToxRp, the second cysteine Cys293 is positioned near the C‐terminus. The C‐terminal region (P271‐E294) including C293 is not assignable in the ^15^N‐HSQC due to motions in intermediate exchange on the NMR time scale (0.5 ms to 1 s) resulting in extensive broadening of the signals. It does not form stable secondary structure elements in solution under the applied conditions, and therefore, produces no observable Nuclear Overhauser Effects (NOEs).

We previously reported that the cysteines in ToxRp are in an oxidized form using a 2,2,2‐trifluoroethyl 6‐thio‐β‐d‐glucopyranoside as a selective tag for cysteines (Fröhlich et al., 2012). This is confirmed by the Cβ chemical shift of C236. Therefore, the presented NMR structure represents the monomeric oxidized form of ToxRp (ToxRp‐ox), which is the active conformation of ToxR in vivo (Fengler et al., 2012; Lembke et al., 2018; Mey et al., 2012; Ottemann and Mekalanos, 1996). This is supported by recently published data on a ToxR homolog from *Vibrio vulnificus* (amino acid sequence identity 55.2%) which suggested that the protein lacks a dimerization interface and therefore forms an intramolecular disulfide bond under nonreducing conditions (Midgett et al., 2020).

### 2.2 The hydrophobic core of ToxRp‐ox forms around the α‐helix

The positioning of the helix of ToxRp‐ox is stabilized by its hydrophobic network (Figure 2d). Half of the helix is buried in the interior of the protein with residues L230, I233, V237, and Y240 involved in the formation of the hydrophobic core.

L226 is located close to the N‐terminus of the helix and interacts together with I233 from the helix with L209 from strand1. I233 from the helix is interacting with V217 of strand2. V237 from the helix, close to activity essential C236, builds apolar contacts to all four beta strands, and is therefore most probably highly significant for the fold. In detail, it contacts V212 from strand 1, V215 and V217 from strand 2, V253 from strand3 and L265 from strand4. Y240 from the helix forms π‐π stacking forces with Y267 from strand4. The β strands form a four stranded β sheet which is additionally stabilized by hydrophobic networks between the strands. Strand 1 forms hydrophobic contacts with L209 to V217 of strand 2, and additionally to V212 to V215 also from strand 2. V215 and V217 from strand 2 are close to V253 from strand 3. V253 interacts with L265 and Y267 from strand 4. Strand 3 is mostly stabilized by V253.

### 2.3 Two conformations of ToxRp are found under reducing conditions

**FIGURE 3:**
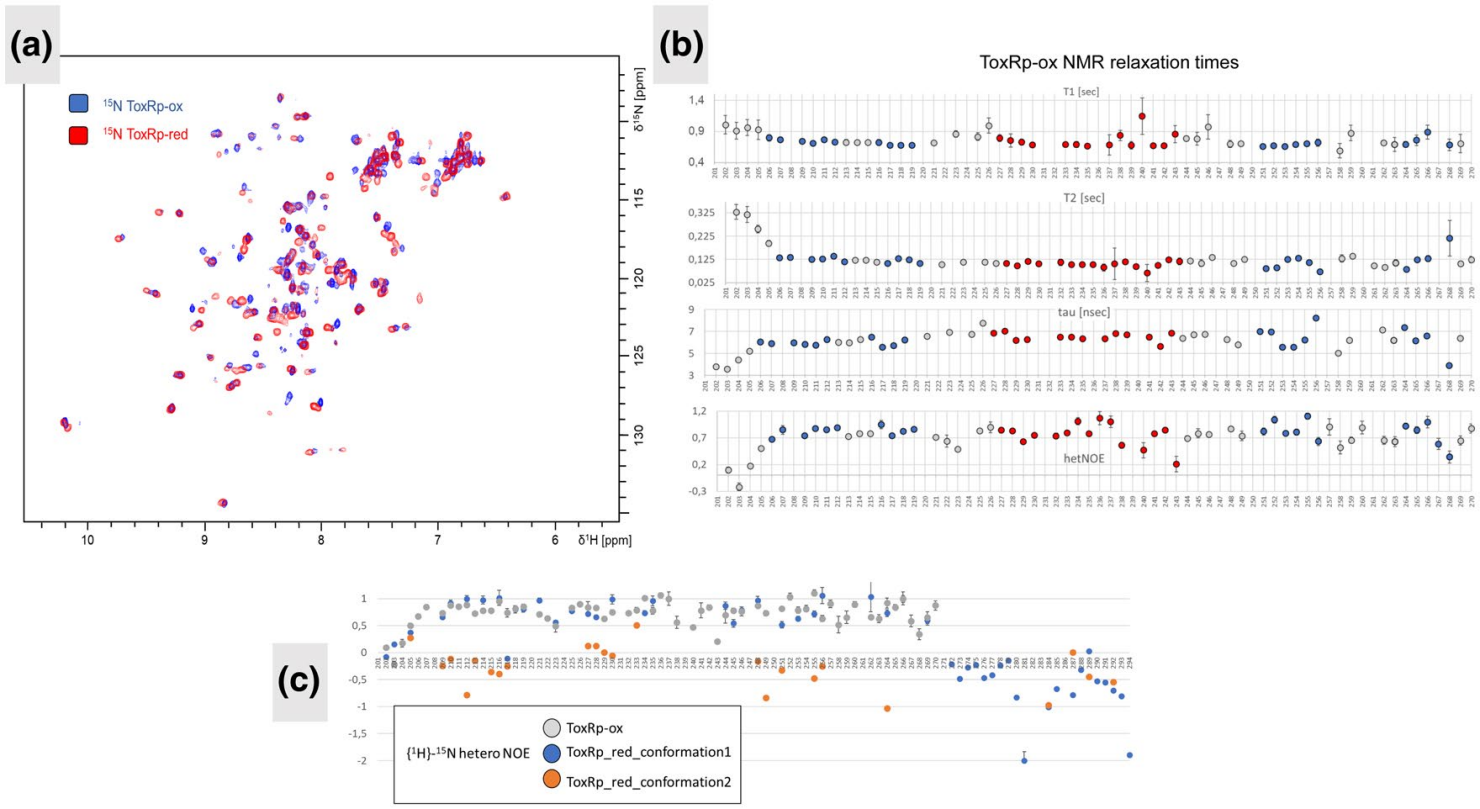
Comparison of ToxRp‐ox and ToxRp‐red. (a): Overlay of ^15^N‐HSQC spectra of ToxRp with reducing agents (red) and without (blue). The spectra can be aligned nicely revealing that under reducing conditions ToxRp‐red has a similar fold compared to ToxRp‐ox. Nevertheless, additional peaks appear in the middle region of the spectrum, revealing a second unstructured conformation. (b): NMR relaxational studies of ToxRp‐ox revealing flexible N‐ and C‐terminal regions. The overall rotational correlation time τ is 6ns, confirming the monomeric state of the protein. (c): {^1^H}^‐^^15^N hetero NOE values of ToxRp‐ox (grey) compared to ToxRp‐red. ToxRp‐red adapts two conformations: one conformation resembles the fold of ToxRp‐ox (blue), the second conformation shows increased flexibility and reveals peaks in the unstructured region of the ^15^N‐HSQC (orange). The C‐terminal stretch (P271‐E294) is only visible in the spectrum when reducing agents are added

Under reducing conditions, ToxRp adapts two conformations accompanied by a second set of peaks in the backbone spectra (Figure 3a). One conformation resembles the ToxRp‐ox structure closely. The second conformation shows signals in the typical random coil regions of ^15^N‐HSQC spectra. The amount of the two conformations of ToxRp under reducing conditions is similar, does not change over time and is found repeatedly in independent protein preparations. Therefore, proteolysis can be ruled out. Interestingly, the C‐terminal stretch that could not be assigned under oxidative conditions, is visible in the NMR spectra recorded under reducing conditions adapting an unstructured and highly flexible conformation. Noteworthy, we cannot exclude that the C‐terminus has a second ‘NMR‐invisible’ conformation moving on an intermediate time scale.

The presence of reducing agents has a significant effect on the ToxRp structure, especially on its C‐terminal region containing the second cysteine. The presence of a second unstructured conformation visible in the NMR spectra under reducing conditions indicates that the reduction of the disulfide bridge destabilizes the ToxRp fold. Indeed, previous in vivo studies could show a higher instability of reduced ToxRp (Lembke et al., 2020; Lembke et al., 2018). It was hypothesized that the activity of ToxR is controlled by its degradation and stability, which itself is dependent on the redox state of the cysteines in the periplasmic domain. A comparison of ^15^N‐HSQC spectra of ToxRp with and without reducing agents (Figure 3a) shows that the structured conformation of ToxRp‐red is similar to ToxRp‐ox, which forms an intramolecular disulfide bond. The absence of a disulfide bond does not change the overall fold of the protein, but significantly alters its stability. This is corroborated by the Talos + secondary structure prediction, using the backbone assignments, that shows a similar pattern of secondary structure for both monomeric redox states: an alpha‐helix, flanked by two beta strands on each side (Figure S1).

### 2.4 ToxRp‐ox shows a well‐structured fold in the N‐terminal domain, with a flexible C‐terminus

To gain further insight into the dynamical features of ToxRp in oxidized and reduced form, we carried out ^15^N T_1_ and T_2_ relaxation as well as {^1^H}‐^15^N NOE measurements (Figure 3b). These data of ToxRp‐ox reveal variations of internal dynamics across the protein chain. The N‐terminal part, which is located close to the inner membrane in vivo, as well as the C‐terminal stretch, show a higher flexibility. ToxRp‐ox contains a well‐structured core flanked by these dynamic regions. The flexibility of the protein increases closer to the C‐terminus. The C‐terminal beta strand shows a higher flexibility than the N‐terminal strands, which indicates that the following C‐terminal stretch might very well be more dynamic. The overall rotational correlation time of ToxRp‐ox is around 6 ns. The molecular weight of the protein can be estimated by the formulation (II) described in Materials and Methods. According to the calculation the rotational correlation time resembles to a well‐structured 12 kDa protein, thereby fitting to a monomeric form of ToxRp‐ox.

Relaxational studies under reducing conditions reveal the dynamic differences of the two conformations formed in the absence of the disulfide bridge (Figure 3c). One conformation seems to adapt a similar fold compared to ToxRp‐ox as shown in the Talos + prediction. A comparison of the {^1^H}‐^15^N hetero NOE values of both redox states show that they also have similar internal dynamics in the N‐terminal region (Figure 3c). The C‐terminus, however, is highly flexible in the absence of the disulfide bond, thereby resulting in strong negative {^1^H}‐^15^N hetero NOEs. The second conformation formed by ToxRp under reducing conditions shows low to negative {^1^H}‐^15^N hetero NOE values indicating strong flexibility, probably due to a loss of structure.

### 2.5 The periplasmic domains of *V. cholerae* ToxR and ToxS forming a strong salt‐dependent heterodimer (ToxRSp) independent on the redox state of ToxR cysteines

Despite its essential function, ToxS remains one of the least characterized proteins in the ToxR regulon (Miller et al., 1989). We were able to express the periplasmic domain of ToxS (ToxSp), whose gene was chemically synthesized in *E. coli* codon optimized form, and record NMR spectra revealing its instable structure. In the absence of its interaction partner ToxRp, ToxSp tends to aggregate rapidly. The ^15^N‐HSQC spectrum of the protein reveals very broad peaks indicative of unspecific aggregation or flexibility. Furthermore, additional peaks appear over time resulting from the degradation of the C‐terminus.

**FIGURE 4:**
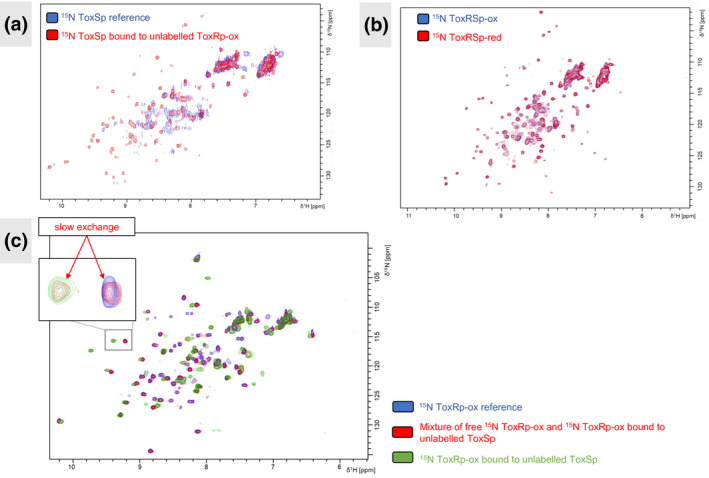
NMR experiments with the heterodimer ToxRSp. (a): Overlay of ^15^N labelled ToxSp alone (blue) and in complex with ToxRp‐ox (red). Formation of the ToxRSp complex stabilizes ToxSp structure. (b): Overlay of ^15^N labelled ToxRSp complex with reducing agents (ToxRSp‐red: red) and without (ToxRSp‐ox: blue). The complex formation is not influenced by the redox states of ToxRp cysteines. (c): Overlay of ^15^N‐HSQC spectra of labelled ToxRp‐ox. The spectrum in blue displays pToxR‐ox alone. The red spectrum represents a mixture of free ToxRp‐ox and ToxRp‐ox bound to ToxSp, indicating a slow exchange mechanism. The green spectrum shows the saturated ToxRSp‐ox complex, with labelled ToxRp‐ox

However, when the periplasmic domains of ToxR and ToxS are pooled together a strong stable complex is formed, shown in the spectrum below (Figure 4a). The structure of ToxSp seems to be stabilized by the binding of ToxRp. The hydrodynamic radius determined by the MALS detector leads to a molecular weight of about 28 kDa (Figure S2). This result points to a heterodimer formation of the periplasmic domains of ToxR and ToxS (ToxRSp) consisting of one ToxRp and one ToxSp molecule. Unfortunately, the size and not well‐defined NMR spectra prevent an NMR structure determination of the ToxRp‐ToxSp complex. We performed NMR interaction experiments between ToxSp and ToxRp in oxidized and reduced form, as well as with ToxRp containing cysteine‐to‐serine mutations (ToxRp C236S, ToxRp C293S, ToxRp C236S & C293S) (see Figures S4 and S5). ToxSp interacts with ToxRp independently of the presence or the redox states of the cysteines. The comparison of the spectra furthermore shows that the formed complexes are structurally similar (Figure 4b). Previous in vivo experiments also suggested that ToxS interaction is not dependent on ToxR cysteines, since double cysteine‐to‐serine mutations of ToxR showed transcriptional activity in the presence of ToxS and bile acid (Lembke et al., 2018).

Interestingly, the stability of the ToxRSp complex is highly dependent on the salt concentration. Salt concentrations below 100 mM induce immediate precipitation of the proteins when they are pooled together.

According to the NMR spectra shown in Figure 4c no exchange on the NMR time scale occurs between the free and the bound state of ToxRp when its binding sites are not saturated. The spectrum can be perfectly aligned with the free and the bound form of ToxRp, thereby indicating a slow exchange mechanism usually connected to strong binding events (Becker et al., 2018). The slow exchange mechanism could be detected in all NMR interaction experiments, thereby indicating that the redox state of ToxRp does not influence the binding strength to ToxSp drastically under the measured conditions.

The strong interaction could be confirmed by fluorescence anisotropy experiments by which we determined a *K_D_* of 11.6 ± 3.4 nM (Supplemental Figure 3).

### 2.6 Binding of ToxSp slows down the proteolytic degradation of ToxRp‐ox

**FIGURE 5:**
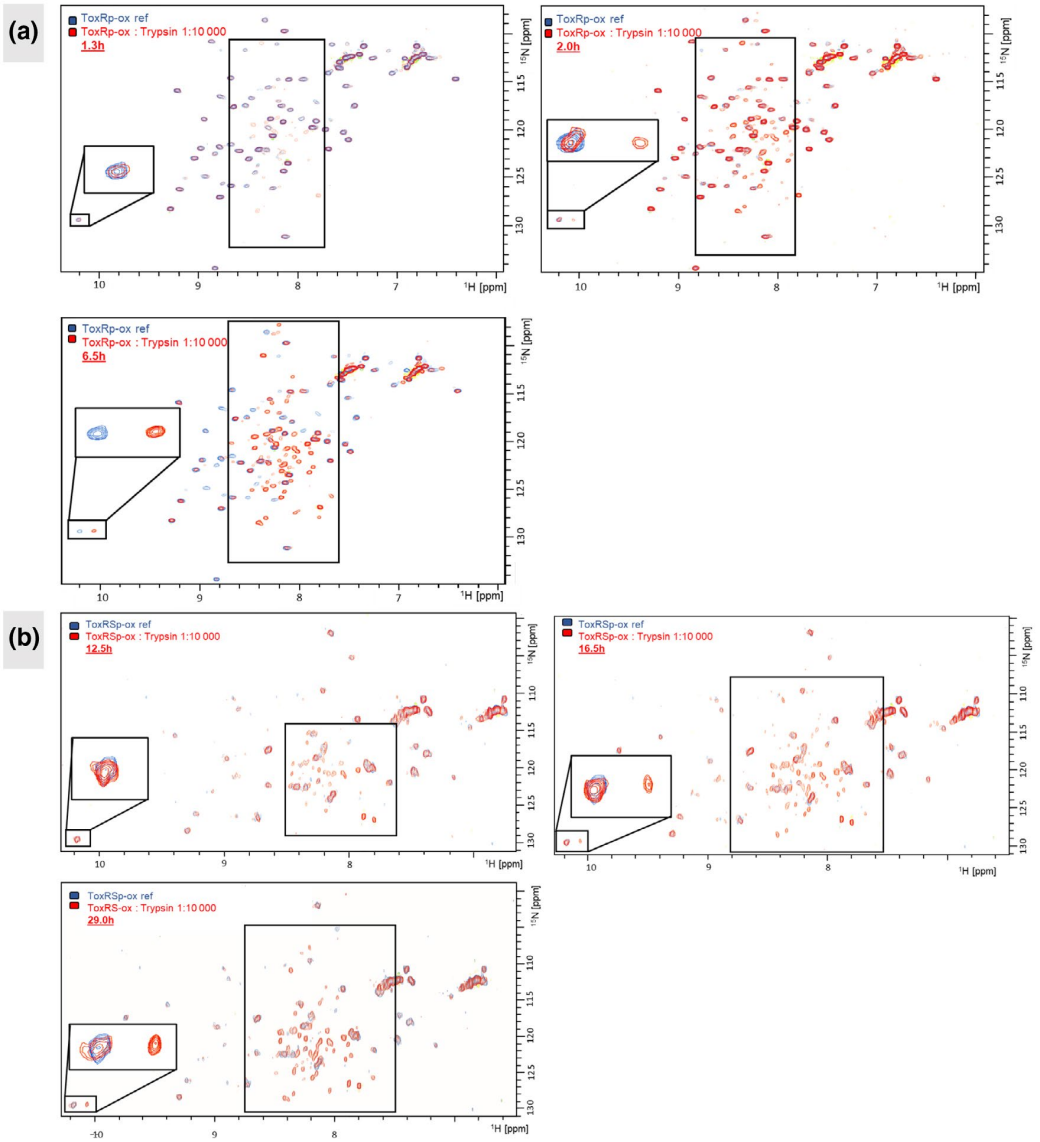
Probing the putative protective function of ToxS by NMR solution experiments. (a): Monitoring the cleavage of ToxRp‐ox with trypsin over time. Sidechain W229, positioned at the N‐terminus of the helix, as well as the middle region of the spectrum are highlighted. After 6.5h the helix is completely digested showing only the unstructured W229 side chain signal. (b): Monitoring the cleavage of the complex of ToxRSp‐ox with trypsin over time. ToxRp‐ox is 15N labelled and ToxSp unlabelled. Sidechain W229, positioned at the N‐terminus of the helix, as well as the middle region of the spectrum are highlighted. The digestion of ToxRp‐ox is protected by ToxSp binding. After 29.0 hr there is still a structured W229 side chain peak visible

ToxS is suggested to protect ToxR from proteolysis (Almagro‐Moreno et al., 2015c; Pennetzdorfer et al., 2019). We monitored ToxRp‐ox digestion by trypsin over time using NMR solution experiments and compared the proteolytic cleavage of ToxRp‐ox alone to ToxRp‐ox in complex with ToxSp. We chose two parameters for estimating the degree of digestion of ToxRp‐ox. One parameter is the chemical shift of the HN side chain peak of W229 which is at the N‐terminal end of the alpha helix of ToxRp‐ox and shows a significant change in the chemical shift when it is not structured. As a second parameter, we analyze the presence of additional peaks in the middle region of the spectrum, where usually unstructured residues appear. After approximately 1.3 hr, we already observe additional signals in the unstructured region of the spectra, nevertheless the helix seems to be still intact (Figure 5a). The next spectrum is recorded after 2.0 hr and shows two peaks for the W229 side chain HN, meaning that both digested and intact helices are present in the sample. The helix is completely digested after 6.5 hr. The signals in the unstructured middle region of the spectra are gaining in intensity over time.

In comparison, the experiments with the ToxRSp‐ox complex show a significant slower degradation of ToxRp‐ox by trypsin (Figure 5b). After 12.5 hr, unstructured HN signals appear in the middle region of the spectrum. Compared to ToxRp‐ox alone, trypsin digestion of ToxRp‐ox starts approximately 11 hr later when it is bound to ToxSp. The unstructured W229 HN side chain peak appears for the first time after 16.5 hr, which is 14.5 hr later compared to ToxRp‐ox alone. The measurement was stopped after 29 hr. The last recorded spectrum still shows two peaks for the W229 HN side chain, meaning that there are still intact helices present in the sample. The experiments clearly show that ToxRp‐ox is more stable against trypsin proteolysis when it is protected by ToxSp. The digestion of ToxRp‐ox by trypsin is slowed down significantly when ToxSp is bound.

## 3 DISCUSSION

The virulence expression system of the cholera causative bacterium *V. cholerae,* namely ToxR‐regulon, represents a highly complex regulation system thereby enabling the bacterium to rapidly switch from a dormant state to a virulent state (Bari et al., 2013). Under virulence inducing conditions, as present in the human small upper intestine tract (Almagro‐Moreno et al., 2015b), the inner membrane regulator ToxR is constitutively expressed, inducing or repressing the transcription of numerous genes in *V. cholerae (*Krukonis et al., 2000 *)*. Its versatile functionality is connected to complex mechanisms in order to control its activity. Environmental signals like bile salts (Provenzano and Klose, 2000) as well as the inner membrane protein ToxS (Lembke et al., 2018), significantly stimulate the activity of ToxR by directly binding to its sensory periplasmic domain (Midgett et al., 2017; Midgett et al., 2020).

Here, we present the NMR solution structure of the ToxR periplasmic domain from *V. cholerae* revealing an αβ‐fold, consisting of a four stranded beta sheet stacked against an alpha helix followed by a flexible C‐terminus (Figure 1). In the absence of reducing agents, the protein domain forms an intramolecular disulfide bond between C236, in the middle of the helix, and C293 at the C‐terminus, referred to as ToxRp‐ox (Figures 1 and 2).

### 3.1 ToxRp homologs share similarities in the hydrophobic core but adapt different C‐terminal conformations

**FIGURE 6:**
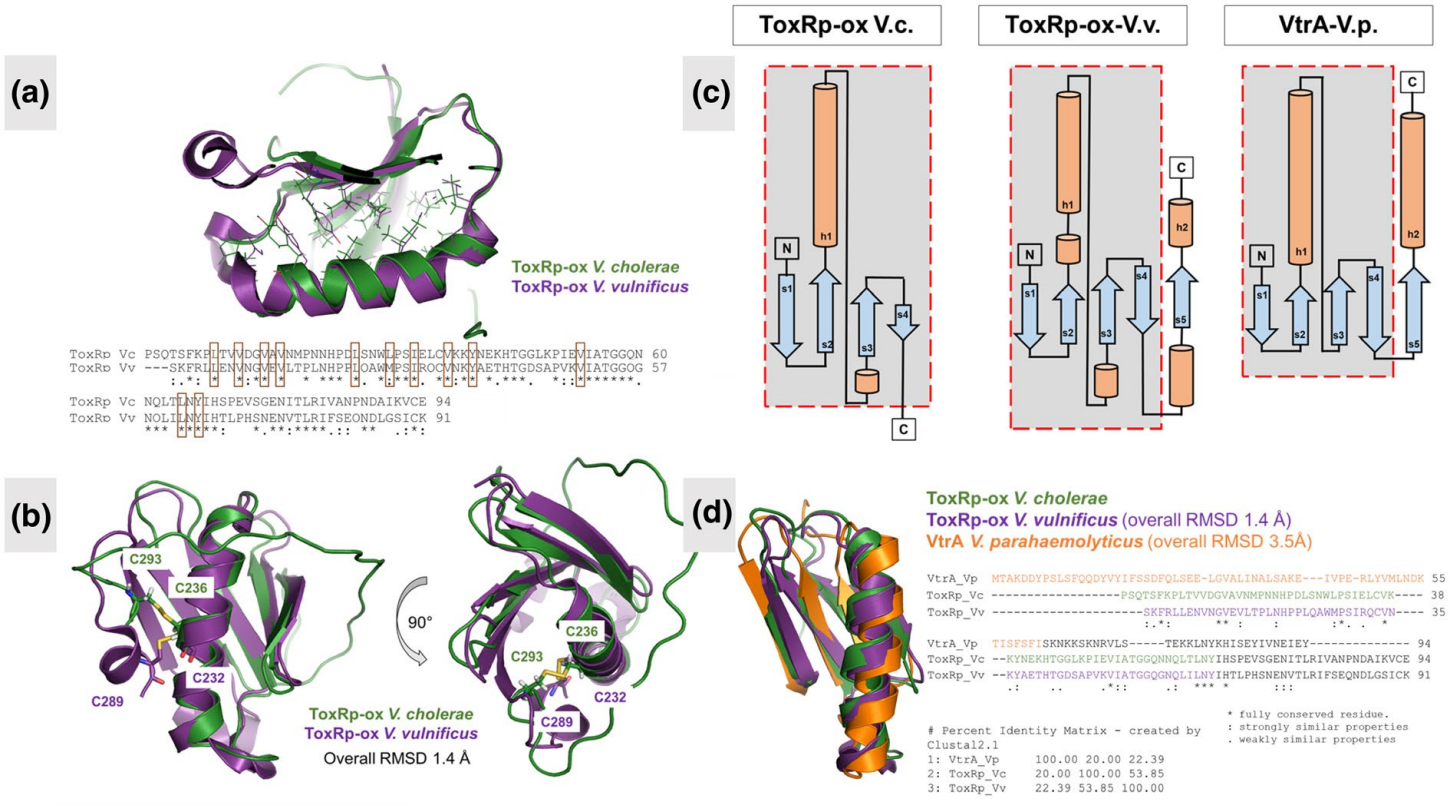
Structural comparison between *V. cholerae* ToxRp‐ox, ToxRp‐ox‐V.v (Midgett et al., 2020) and the VtrA‐V.p. crystal structure in complex with VtrC‐V.p. (Li et al., 2016). (a): Superposition of *Vibrio cholerae* ToxRp‐ox (green) and ToxRp‐ox‐V.v. (purple). Residues shown in sticks are contributing to the hydrophobic core of the proteins. The sequence alignment indicates a conservation of the residues forming the hydrophobic interior (highlighted in brown in the sequence and shown as sticks in the structure). Pi stacking forces stabilize the positioning of the helix (shown in zoomed region). (b): Superposition of *V. cholerae* ToxRp‐ox (green) and ToxRp‐ox‐V.v. (purple). The cysteine residues are forming a disulphide bond and are shown in sticks. The overall RMSD of the structures is 1.4 Å. The structures show significant similarities at the N‐terminus which forms four beta strands and a helix. The C‐terminus of ToxRp‐ox‐V.v. forms an additional beta‐strand and a short C‐terminal helix, in contrast to the unstructured C‐terminus of *V. cholerae* ToxRp‐ox. (c): Topology diagrams of VtrA‐V.p., ToxRp‐ox‐V.v. and *V. cholerae* ToxRp‐ox. The sensory bile acid binding proteins reveal a similar N‐terminal topology comprised by an alpha‐helix flanked by two hairpins on each side. The C‐terminal region shows structural differences for each protein. (d): Structural and sequential alignment (excluding the C‐terminal regions) of *V. cholerae* ToxRp‐ox (ToxRp‐ox‐V.c.) (green), ToxRp‐ox‐V.v. (purple) and VtrA‐V.p (orange). The proteins share a similar N‐terminal fold, comprised by a helix stacked against a four stranded beta sheet. The residues shown in the cartoon structure are highlighted in the sequence

A comparison of the residues involved in the stabilization of the core structure of *V. cholerae* ToxRp‐ox and *V. vulnificus* ToxRp‐ox‐V.v. (Midgett et al., 2020) show significant similarities in the amino acids and their positioning in the structure (Figure 6a). This could indicate that the hydrophobic core of ToxR is conserved in the *Vibrio* family.

Regarding the C‐terminal region the structures of ToxRp‐ox from *V. cholerae* and *V. vulnificus* significantly differ (Figure 6b). ToxRp‐ox‐V.v. has an additional beta strand (β5) and an additional short helix (α2) in the C‐terminal region (Midgett et al., 2020). The C‐terminal stretch following beta strand 4 is invisible in the NMR spectra of *V. cholerae* ToxRp‐ox, and thus, does not develop a stable NMR‐observable structure. We propose that the cysteine‐cysteine connection confines its motion leading to an intermediate exchange on the NMR time scale. Since the C‐terminus is involved in the activity dependent formation of the disulfide bridge, it is possible that the C‐terminal region differs among *Vibrio* species and may be essential for their individual functions. Another explanation for the differences observed in the C‐termini could be the different physicochemical states under which the structures are determined. It is also possible that the C‐terminal region has a low tendency to form structural elements, which under solution conditions is not strong enough to result in a stable folding. In a solid form, crystal packing forces may artificially stabilize the formation of these secondary structure elements in the C‐terminus.

### 3.2 V. cholerae ToxRp‐ox, ToxRp‐ox‐Vibrio vulnificus (ToxRp‐ox‐V.v.), and VtrA‐Vibrio parahaemolyticus (VtrA‐V.p.) share a similar N‐terminal fold

A comparison of *V. cholerae* ToxRp‐ox and ToxRp‐ox‐V.v. (Midgett et al., 2020) to the VtrA‐V.p. crystal structure in complex with VtrC‐V.p. (Li et al., 2016) shows that all three sensory bile acid binding *Vibrio* proteins share the same N‐terminal fold comprised by a helix stacked against a four stranded beta sheet (Figure 6c,d). Although the proteins do not show a high sequence identity, they reveal a significant structural similarity in this region. The overall RMSD between the N‐terminal regions of ToxRp‐ox from *V*. *cholerae* and ToxRp‐ox‐V.v. is 1.4 Å, between ToxRp‐ox and VtrA‐V.p. 3.5Å. This fold may be connected to their signaling activity including the binding to bile acid.

### 3.3 A reduction of ToxRp cysteines lead to a destabilization of its structure

To address the question of the role of the redox state on the structure of ToxRp, we assigned the backbone of ToxRp under reducing conditions (ToxRp‐red) and could detect that ToxRp adapts two conformations when its cysteines are reduced (Figure 3c). The first conformation is structurally similar to the monomeric oxidized form of ToxRp, the second one is highly flexible and probably unstructured. The disruption of the intramolecular disulfide bond therefore seems to destabilize the ToxRp fold.

The C‐terminal stretch of ToxRp is only visible in the ^15^N‐HSQC NMR spectra when reducing agents are added, exhibiting fast dynamics. Thus, the movement of the C‐terminus is restricted when the disulfide bridge is formed, leading to broadening of the signals due to intermediate exchange on the NMR chemical shift time scale.

Previous studies reported an increased degradation of ToxR under reducing conditions (Fengler et al., 2012; Lembke et al., 2018; Pennetzdorfer et al., 2019). The presented NMR experiments show that the dynamic C‐terminus of ToxRp indeed plays a significant role in the stability of the ToxRp structure. A reduction of the cysteines induces a second unstructured flexible conformation of ToxRp and furthermore increases the dynamics of the C‐terminus. This could be an explanation for the more effective in vivo cleavage of reduced ToxR by periplasmic proteases DegPS (Lembke et al., 2018). One possibility for regulating ToxR activity could therefore be via controlling the redox state of the cysteines of the ToxR periplasmic domain.

### 3.4 ToxRp interacts with ToxSp in a 1:1 fashion resulting in a strong heterodimer

There exist still many unanswered questions regarding the mechanism of the virulence essential ToxRS interaction in *V. cholerae*. Our experiments reveal a heterodimer formation between the periplasmic domains of ToxR and ToxS (Figure 4). Interestingly, the binding event is independent of the redox state of the cysteines of ToxRp. Also, the complexes formed between ToxSp and reduced or oxidized ToxRp, as well as ToxRp containing a cysteine to serine mutation (Figures S4 & S5), are structurally similar. The slow exchange mechanism visible in all NMR interaction experiments reveal that strong binding occurs independent of ToxRp cysteine oxidation states. The dissociation constant of 11.6 nM determined by fluorescence anisotropy experiments, confirms the formation of a stable strong complex (Figure S3).

Although the binding of ToxS is not affected by the redox state of ToxR cysteines, its stability is highly dependent on the salt concentration. The ToxRSp complex tends to aggregate immediately under salt concentrations below 100 mM, indicating that hydrophobic and electrostatic interactions play a key role in this binding event.

In addition, NMR trypsin digestion studies could directly show a significant slowing down of the degradation of ToxRp when it is bound in the heterodimer complex (Figure 5). ToxSp seems to protect putative protease cleavage sites of ToxRp and thereby increases its stability. We propose that the interaction between ToxR and ToxS is another mechanism to control the activity of ToxR by altering its accessibility to proteases. The ToxRS control‐mechanism works independently of its cysteines but is dependent on the salt concentration.

**FIGURE 7:**
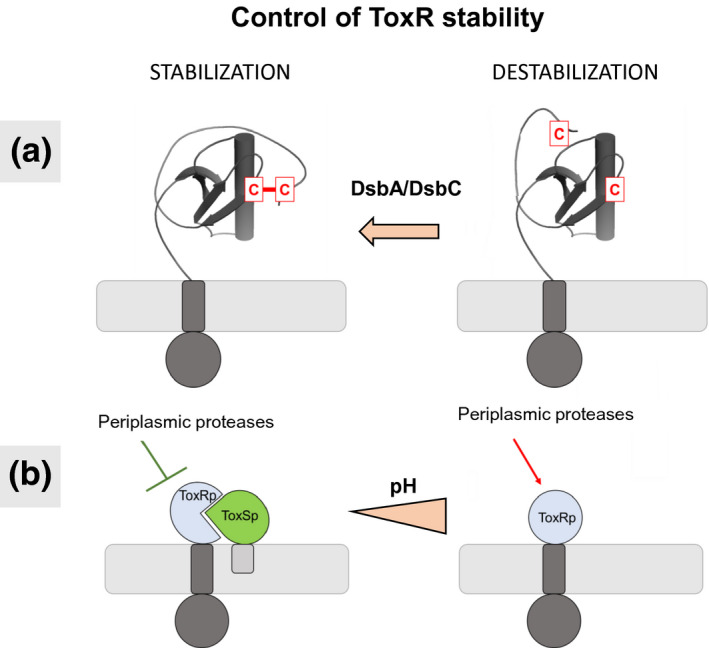
Model of two control mechanism for ToxR activity. (a): Reduction of ToxRp cysteines leads to a destabilization of the structure. The redox state of ToxR depends on DsbA/DsbC invivo (Fengler et al., 2012; Lembke et al., 2018; Lembke et al., 2020) (b): Formation of a heterodimer between the periplasmic domains of inner membrane proteins ToxR and ToxS, protects ToxR from rapid proteolytic cleavage. Alkaline pH decreases ToxRS interaction and subsequently leads to ToxR proteolysis (Midgett et al., 2017)

Since ToxR has versatile functions and can act as activator, co‐activator or repressor, it is likely that its activity is also regulated on different levels. We propose that ToxR activity is mainly controlled by its stability which is dependent on different factors (Figure 7). The reduction of ToxRp cysteines represent one possibility to decrease ToxR stability. This regulation is controlled by periplasmic oxidoreductases DsbA and DsbC *in vivo* (Fengler et al., 2012; Lembke et al., 2020; Lembke et al., 2018). The interaction with ToxS represents another possibility to increase ToxR stability by directly protecting ToxRp from proteases DegPS (Lembke et al., 2018; Pennetzdorfer et al., 2019). Binding of ToxS seems to be independent from ToxRp cysteines. Previous work has shown that *V. cholerae* alkalinizes its surrounding in the late stationary phase, which decreases the interaction between ToxRS and subsequently leads to a loss of function of ToxR due to proteolysis (Almagro‐Moreno et al., 2015a; Midgett et al., 2017). Furthermore, our experiments show that binding of ToxSp does not trigger dimerization of ToxRp via disulfide bonds under the applied conditions. Therefore, our data also support the theory that dimerization of ToxR, in order to induce transcription, is activated by the presence of DNA (Lembke et al., 2020; Midgett et al., 2020).

## 4 METHODS

### 4.1 Cloning expression purification

All constructs listed in Table 1 were generated by using standard procedures and verified via automated sequencing. For protein expression *E. coli* BL21 DE cells were used. Cells were grown under 180 rpm and induced with 1 mM IPTG when OD_600_ reached 0.6 to 0.8. ToxRp producing cultures were incubated at 37°C overnight, ToxSp was expressed at 37°C before induction, after induction at 20°C overnight. For production of non‐isotopically labeled proteins LB media was used, for isotopic labeled proteins cells were grown in M9 minimal media containing ^15^N labeled (NH_4_)2SO_4_ and ^13^C labeled glucose.

**TABLE 1:** List of constructs that are used in the described experiments including the amino acid sequence of each protein domain

Name	Vector	His‐tag plus AA sequence
ToxRp	pQE30	MRGSHHHHHHGSPSQTSFKPLTVVDGVAVNMPNNHPDLSNWLPSIELCVKKYNEKHTGGLKPIEVIATGGQNNQLTLNYIHSPEVSGENITLRIVANPNDAIKVCE
ToxRp C236S	pQE30	MRGSHHHHHHGSPSQTSFKPLTVVDGVAVNMPNNHPDLSNWLPSIELSVKKYNEKHTGGLKPIEVIATGGQNNQLTLNYIHSPEVSGENITLRIVANPNDAIKVCE
ToxRp C293S	pQE30	MRGSHHHHHHGSPSQTSFKPLTVVDGVAVNMPNNHPDLSNWLPSIELCVKKYNEKHTGGLKPIEVIATGGQNNQLTLNYIHSPEVSGENITLRIVANPNDAIKVSE
ToxRp C236S & C293S	pQE30	MRGSHHHHHHGSPSQTSFKPLTVVDGVAVNMPNNHPDLSNWLPSIELSVKKYNEKHTGGLKPIEVIATGGQNNQLTLNYIHSPEVSGENITLRIVANPNDAIKVSE
ToxSp	pCDFDuet	MGSSHHHHHHSQDPNSDFKLEQVLTSREWQSKMVSLIKTNSNRPAMGPLSRVDVTSNVKYLPNGTYLRVSIVKLFSDDNSAESVINISEFGEWDISDNYLLVTPVEFKDISSNQSKDFTDEQLQLITQLFKMDAQQSRRVDIVNERTILF TSLSHGSTVL FSNS
ToxSp H10_S11insC	pCDFDuet	MGSSHHHHHHCSQDPNDFKLEQVLTSREWQSKMVSLIKTNSNRPAMGPLSRVDVTSNVKYLPNGTYLRVSIVKLFSDDNSAESVINISEFGEWDISDNYLLVTPVEFKDISSNQSKDFTDEQLQLITQLFKMDAQQSRRVDIVNERTILF TSLSHGSTVL FSNS

After overnight expression, cell cultures were centrifuged, and the pellet dissolved in 20 ml of loading buffer containing either 8 M urea, 300 mM sodium chloride, 10 mM imidazole pH 8 for ToxRp constructs, or 20 mM Tris, 300 mM sodium chloride, 10 mM imidazole pH 8 for ToxSp constructs. Protease inhibitor was added to the loading buffer. Reduction of the cysteines was achieved via addition of 4 mM 2‐mercaptoethanol (β‐ME) to all buffers. The cells were disrupted via sonication. The lysate was centrifuged, and the supernatant was loaded on a gravity column containing 2 ml of Ni‐NTA agarose beads. The column was washed with 15 column volumes CV of loading buffer followed by 5 CV of the loading buffers containing 1 M sodium chloride and 5 CV of the loading buffer containing 20 mM imidazole. His‐tagged proteins were eluted with 5 CV elution buffer containing 330 mM imidazole. ToxRp constructs were refolded overnight by dialysis at 4°C in 50 mM sodium phosphate, 300 mM sodium chloride, pH 8, with or without 4 mM β‐ME. The final purification step includes purification by FPLC using a HiLoad 26/600 Superdex 75 pg column in 50 mM sodium phosphate, 300 mM sodium chloride, pH 8 with or without 4 mM β‐ME.

### 4.2 NMR experiments

All NMR spectra were recorded on a Bruker Avance III 700 MHz spectrometer equipped with a cryogenically cooled 5 mm TCI probe using z‐axis gradients at 25°C. All NMR samples were prepared in 90% H_2_O/10% D_2_O. Spectra were processed with NMRPipe (Delaglio et al., 1995).

### 4.3 NMR structure ToxRp‐ox

The ToxRp‐ox NMR samples were dissolved in 50 mM sodium phosphate, 100 mM sodium chloride at pH 6.5 and measured in a 5 mm tube. The concentration of the sample was 500 µM. For the assignment of the backbone resonances standard triple resonance experiments were used: HNCO (Grzesiek and Bax, 1992; Kay et al., 1994) , HN(CA)CO (Clubb et al. 1992; Kay et al., 1994), HNCACB (Muhandiram and Kay, 1994; Wittekind and Mueller, 1993), HN(CO)CA (Grzesiek and Bax, 1992; Kay et al., 1994), HNCA (Grzesiek and Bax, 1992; Kay et al., 1994; Schleucher et al., 1993), HN(CA)CO (Clubb et al. 1992; Kay et al., 1994), ^15^N HSQC (Davis et al. 1992). For side‐chain resonance assignments, we used: HCCH‐TOCSY (Bax et al., 1990; Kay et al., 1993; Olejniczak et al., 1992), HCCCONH (H‐detected) (Löhr and Rüterjans, 2002; Montelione et al., 1992), and HCCCONH (C‐detected) (Löhr and Rüterjans, 2002; Montelione et al., 1992). In order to reduce water signals ^13^C NOESY experiments (Davis et al. 1992) were performed in 100% D_2_O. For that matter, the protein sample was first lyophilized and subsequently dissolved in 100% D_2_O to a final protein concentration of about 0.5 mM. The backbone and side‐chain resonance assignments were carried out with CcpNMR 2.4.1. (Skinner et al., 2017). For the structure calculation CYANA (3.98.5) (Güntert et al., 1997; Herrmann et al., 2002) was used. NOESY peaks were manually picked and automatically assigned using CYANA (3.98.5) (Güntert et al., 1997; Herrmann et al., 2002). The final structure ensemble, consisting of 20 conformers, was validated by PSVS (Bhattacharya et al., 2007), molecular images were created using PyMOL (Delano Scientific, (The PyMOL Molecular Graphics System, Version 1.2r3pre, Schrödinger, LLC)). Secondary structure predictions were done using backbone assignments and TALOS+ (Shen et al., 2009).

### 4.4 Backbone assignment ToxRp‐red

NMR spectra of 500 µM ToxRp‐red were recorded in a 5 mm tube dissolved in 50 mM sodium phosphate, 100 mM sodium chloride, 4 mM DTT at pH 6.5. For the assignment of the backbone resonances standard triple resonance experiments were used: HNCO (Grzesiek and Bax, 1992; Kay et al., 1994), HN(CA)CO (Clubb et al. 1992; Kay et al., 1994), HNCACB (Muhandiram and Kay, 1994; Wittekind and Mueller, 1993), HN(CO)CA (Grzesiek and Bax, 1992; Kay et al., 1994), HNCA (Grzesiek and Bax, 1992; Kay et al., 1994; Schleucher et al., 1993), HN(CA)CO (Clubb et al. 1992; Kay et al., 1994), and ^15^N HSQC (Davis et al. 1992). The backbone assignments were carried out with CcpNMR 2.4.1. (Skinner et al., 2017). Secondary structure predictions were done using backbone assignments and TALOS+ (Shen et al., 2009).

### 4.5 NMR interaction experiments

For studying the ToxRp/ToxSp interaction ^15^N HSQC (Davis et al. 1992) experiments were recorded. Interaction experiments with ^15^N labeled ToxRp and unlabeled ToxSp were recorded using a buffer containing 50 mM sodium phosphate, 100 mM sodium chloride at pH 6.5. Interaction experiments with ^15^N labeled ToxSp and unlabeled ToxRp were carried out in a buffer containing 20 mM Bis Tris, 100 mM Sodium chloride at pH 7. For achieving a reduction of cysteines 4 mM DTT was added to these samples.

### 4.6 NMR relaxation experiments

NMR relaxation experiments of ToxRp‐ox and ‐red were recorded in a buffer containing 50 mM sodium phosphate, 100 mM sodium chloride at pH 6.5, with or without 4 mM DTT. ^15^N T_1_ values were measured using hsqct1etf3gpsi3d.2 Bruker pulse program, with delay times of: 0.4, 2.0, 0.2, 1.5, 0.5, 0.7, 1.0, 0.3, 3.0, 3.5, 0.1, 0.8, and 0.05 s. ^15^N T_2_ values were measured using hsqct2etf3gpsi3d with varying delays of 0.68, 0.22, 0.229, 0.034, 0.204, 0.17, 0.114, 0.085, 0.237, 0.136, 0.051, 0.170, and 0.102 s. The {^1^H}‐^15^N heteronuclear NOEs were measured by using the pulse sequence hsqcnoef3gpsi. Spectra were processed using NMRPipe (Delaglio et al., 1995) and analyzed via CcpNMR 2.4.1. (Skinner et al., 2017). The rotational correlation time was calculated using the formula shown below in (I) (Kay et al., 1989). The molecular weight of the protein can be estimated by the formulation determined by the Biomolecular NMR tools from UC San Diego, USA (Brendan Duggan, 2015).

#### 4.6.1 Rotational correlation time

$$\tau c\;\approx \frac{1}{4\pi v_{N}}\sqrt{6\frac{T1}{T2}‐7}$$

τc, rotational correlation time [sec]; *ν_N_*, ^15^N resonance frequency [Hz]; *T*_1_, ^15^N T_1_ relaxation time [sec]; *T*_2_, ^15^N T_2_ relaxation time [sec].

#### 4.6.2 Estimation of the molecular weight via the rotational correlation time

τ=MW×0.433859+0.775137.

τ, rotational correlation time [sec]; MW, molecular weight [kDa].

### 4.7 NMR trypsin degradation study

The trypsin digestion experiments were carried out in 50 mM sodium phosphate, 100 mM sodium chloride at pH 6.5 measured in a 3 mm tube. For the experiments, only ToxRp‐ox was ^15^N‐labeled. The concentration of ^15^N‐labeled ToxRp‐ox and ToxRSp‐ox was 3.14 mg/ml in a volume of 170 µl. A reference 2D ^15^N HSQC was recorded first, then trypsin was added in a 1:10 000 ratio of trypsin to ToxRp‐ox or ToxRSp‐ox. After addition of the protease, a series of ^15^N‐HSQC experiments were acquired.

### 4.8 MALS‐SEC

ToxRp‐ox and ToxSp were purified according to the protocol described before and pooled in a 1:2 ratio with excess of ToxSp. To get rid of unbound ToxSp, the sample was further purified using a Superdex 200 Increase 10/300 column (GE healthcare). The resulting fractions were pooled and concentrated to 0.1 ml, containing 18 mg/ml of ToxRSp‐ox heterodimer using Amicon Ultra Centricons with a 3 kDa cutoff. The sample was loaded on a Superdex 200 Increase 10/300 column (GE healthcare) with a flow rate of 0.5 ml/min performed on a ÄKTA pure 25 (GE healthcare) connected to the miniDAWN Treos II MALS detector (Wyatt). The buffer used for the MALS‐SEC measurement contained 20 mM Tris, 150 mM sodium chloride, pH 7.5. The chromatogram resulted in a single peak eluting after 16.19 ml. The hydrodynamic radius determined by the MALS detector leads to a molecular weight of about 28 kDa, suggesting a heterodimer formation of ToxRSp‐ox.

### 4.9 Fluorescence anisotropy

For the fluorescence anisotropy measurement, a mutant of ToxSp containing a cysteine between the His6x tag and the protein was used. ToxSp H10_S11insC was labeled with the fluorescent dye 5‐iodoacetamidofluorescein 5‐IAF, which reacts to reduced cysteines according to the manufacturers protocol. In short: The mutant was expressed and purified according to the protocol used for ToxSp. A 4 mM β‐ME was added to all buffers to prevent oxidation of the cysteines. ToxSp H10_S11insC was then rebuffered in conjugate buffer (200 mM sodium phosphate, 200 mM sodium Chloride, 1 mM EDTA, and 2 mM β‐ME pH7.5). 5‐IAF was dissolved in DMSO then added in a 10 times excess over ToxSp H10_S11insC and incubated for 2.5 hr in the dark. To remove unbound tags, the sample was purified on a FPLC HiLoad 26/600 Superdex 75 pg column in the dark and dialyzed against to 50 mM sodium phosphate, 150 mM sodium chloride pH 7.2. For the calculation of the concentration of the protein, the formulas shown below (III. and IV.) were used. ToxRp‐ox was expressed and purified according to the previously described protocol.

#### 4.9.1 Correction factor

$$\text{CF}\;=\;\left(A280\right)/\left(\text{Amax}\right).$$

CF, correction factor; A280, Absorption of the protein at 280 nm; Amax, Absorption of the protein at 491 nm.

#### 4.9.2 Protein concentration

$$\left[P\right]\;=\;\left(\text{A280 -}\;\left(\text{Amax}\;\times \text{CF}\right)/\varepsilon \right)\times \;\text{dilution}\;\text{factor}$$

[P], Protein concentration; A280, Absorption of the protein at 280 nm, Amax; Absorption of the protein at 491 nm; CF, correction factor; Ɛ, protein molar extinction coefficient.

The FA measurements were carried out under 25°C on a FP‐6500 spectrofluorimeter equipped with manual excitation and emission polarisers (Jasco, Easton, MD), at an emission wavelength of 525 nm upon excitation at 495 nm, and a high photomultiplier sensitivity. Slits widths are 3 nm and 5 nm for excitation and emission, respectively. The fluorescence anisotropy is defined as (V).

#### 4.9.3 Fluorescence anisotropy

$$r\;=\;\frac{I_{\mathrm{VV}}‐G\;\times I_{\mathrm{VH}}}{I_{\mathrm{VV}}\;+\;2G\times \;I_{\mathrm{VH}}}$$

−0.4 ≤ *r* ≤ 0.4

*R*, Anisotropy; *I_VV_*, Intensity of the vertically polarized component; *I_VH_*, Intensity of the horizontally polarized component; *G*, G‐factor (the instrument sensitivity ratio toward vertically and horizontally polarized light).

where *I_VV_* is the fluorescence intensity recorded with excitation and emission polarisers in vertical positions, and *I_VH_* is the fluorescence intensity recorded with the emission polariser aligned in a horizontal position. The *G* factor is the ratio of the sensitivities of the detection system for vertically and horizontally polarized light *G* = *I_HV_*/*I_HH_*.

The FITC ToxSp H10_S11insC solution is titrated against increasing amounts of ToxRp‐ox diluted in the same buffer. For each point, the anisotropy is recorded over 10 s and the mean r values for each measurement are used. Anisotropy changes were fitted by a Levenberg–Marquardt algorithm to the equation in (VI) (Lakowicz, 2010).

#### 4.9.4 Dissociation constant determination via fluorescence anisotropy

$$rss\;=\;\Delta r_{\max}\frac{K_{d}\;+\;\left[\text{ToxSp}\right]+\left[\text{ToxRp - ox}\right]‐\sqrt{\left(k_{d}+\left[\text{ToxSp}\right]+\left[\text{ToxRp - ox}\right]{}^{2}\right)‐4\left[\text{ToxSp}\right]+\left[\text{ToxRp - ox}\right]}}{2\left[\mathrm{ToxSp}\right]}$$

*r*, Observed anisotropy; *r*_max_, Maximal anisotropy change; K_D_, Dissociation constant.

## Supporting information

Fig S1

Fig S2

Fig S3

Fig S4

Fig S5

Table S1

Supplementary Material
