# Supplementary figures and images for: The periplasmic domains of Vibriocholerae ToxR and ToxS are forming a strong heterodimeric complex independent on the redox state of ToxR cysteines
Source: Mol Microbiol. 2021 Jan 25;115(6):1277–91. doi: 10.1111/mmi.14673 (PMC8359183; doi:10.1111/mmi.14673)

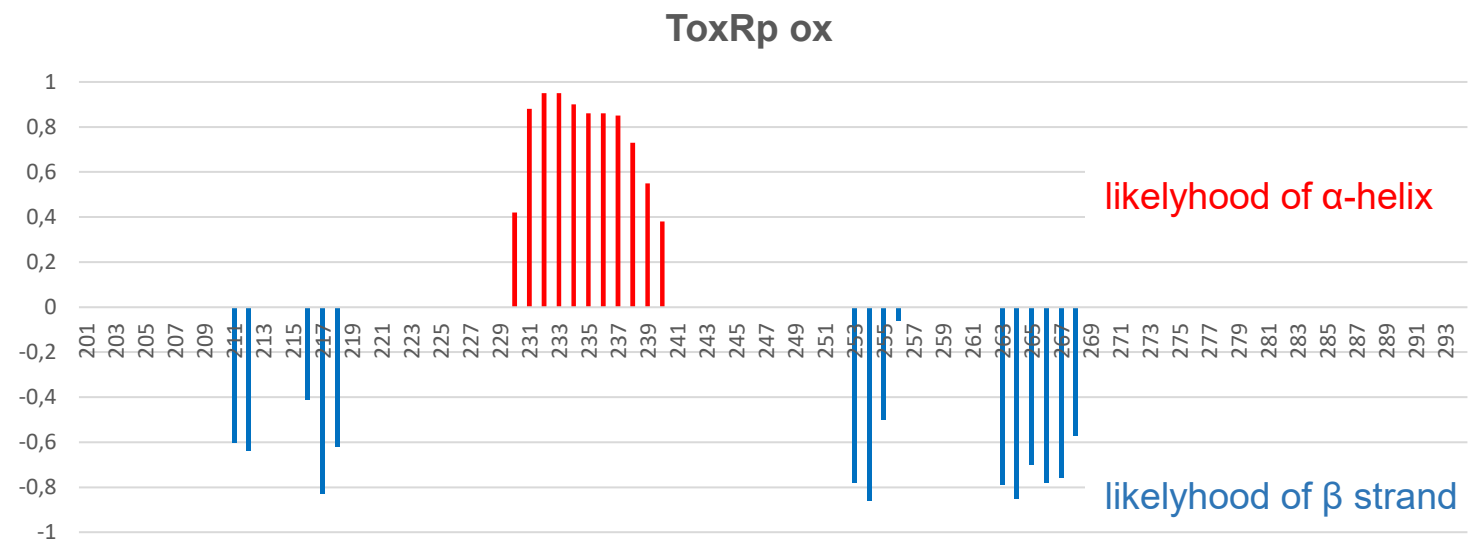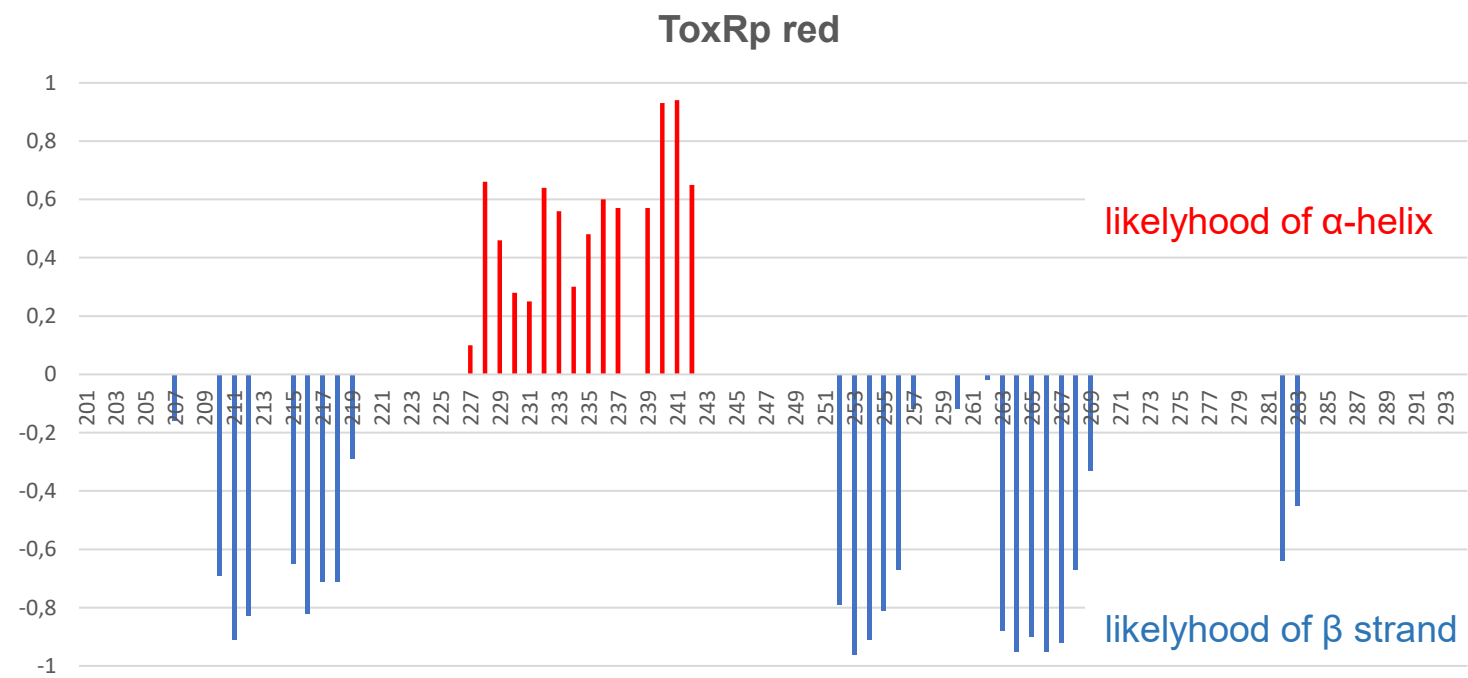

Supplement: Supplementary file 1 — Fig S1 [file MMI-115-1277-s005.pdf]

Molar Mass vs. Volume

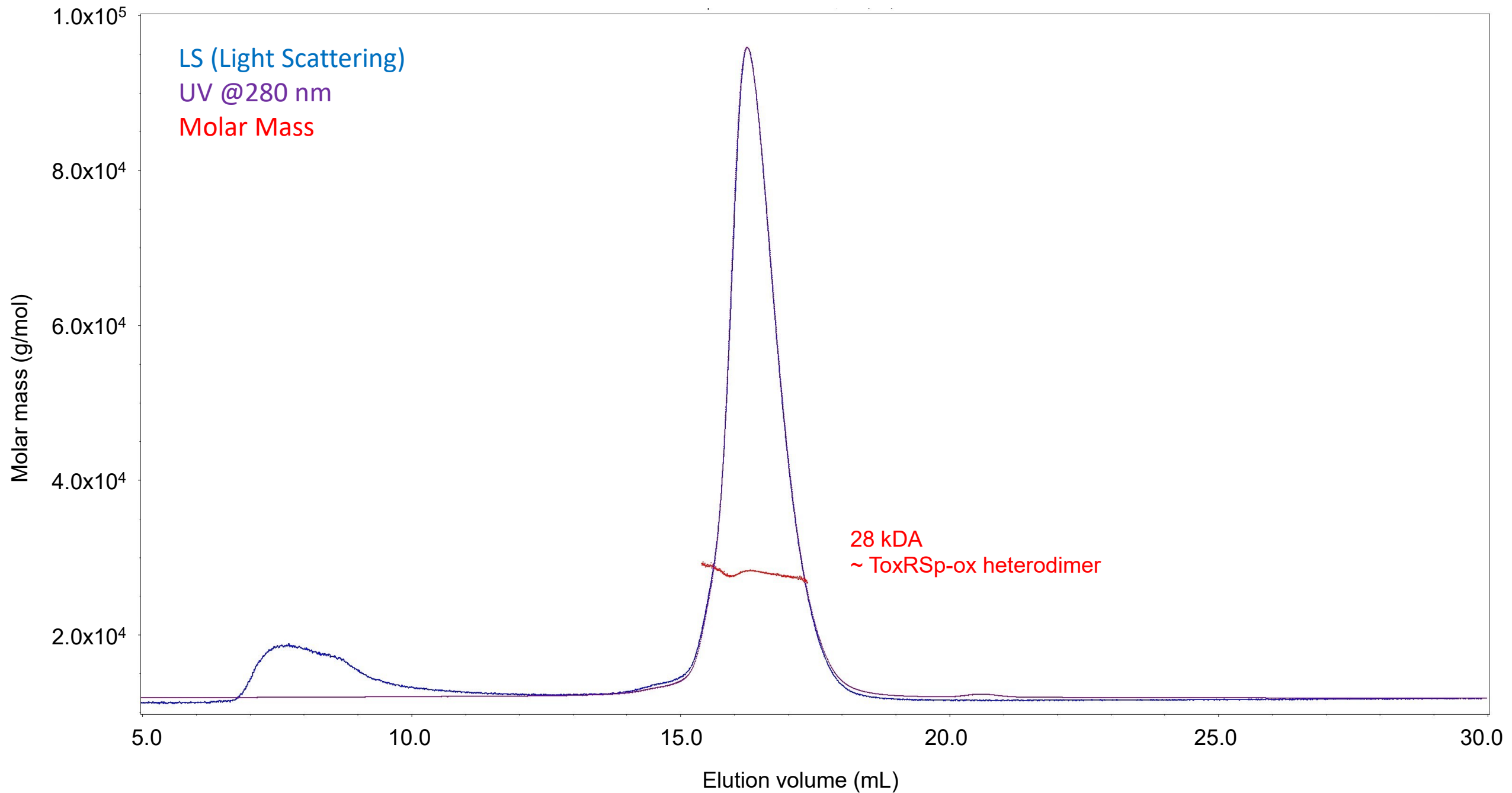

Supplement: Supplementary file 2 — Fig S2 [file MMI-115-1277-s006.pdf]

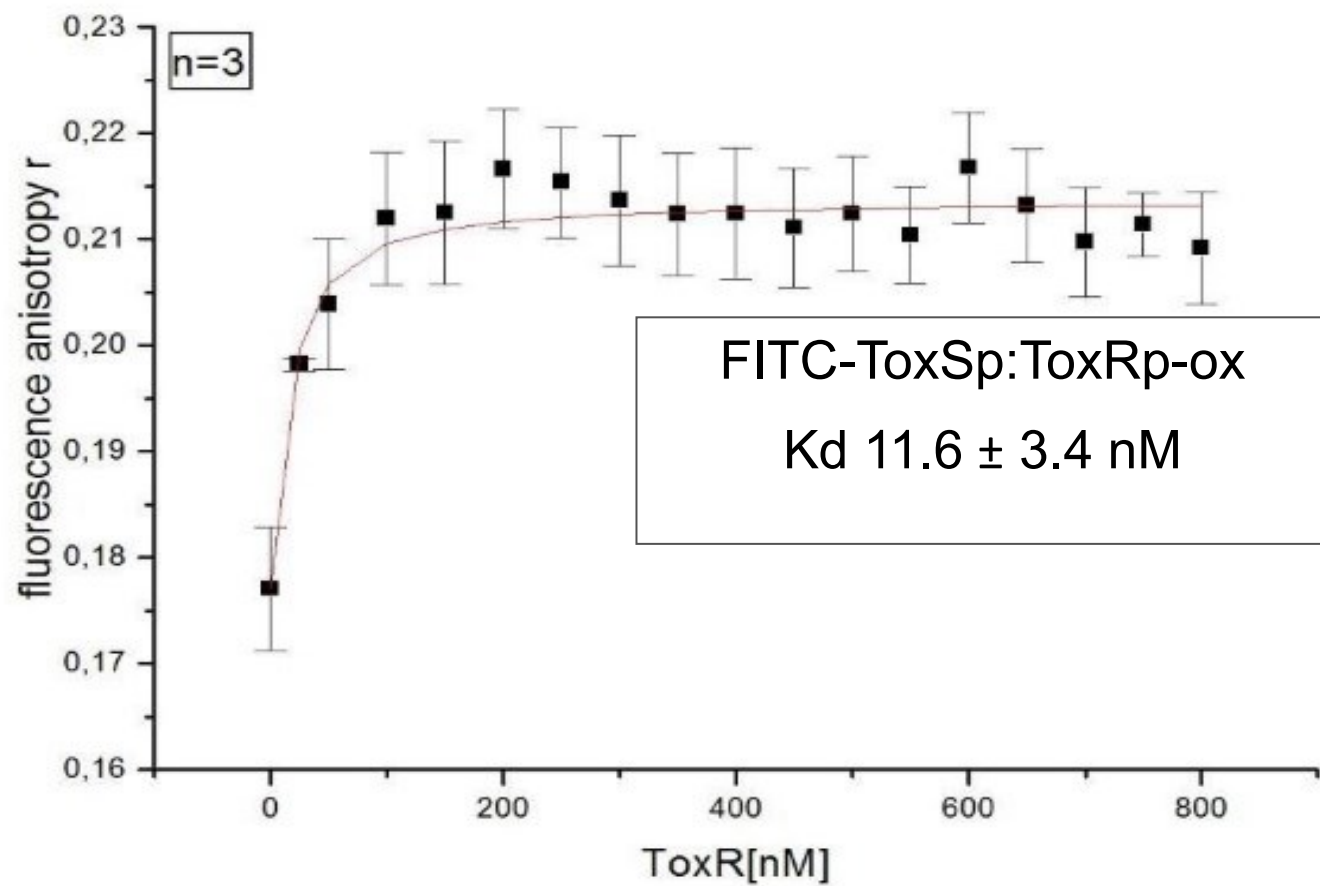

Supplement: Supplementary file 3 — Fig S3 [file MMI-115-1277-s007.pdf]

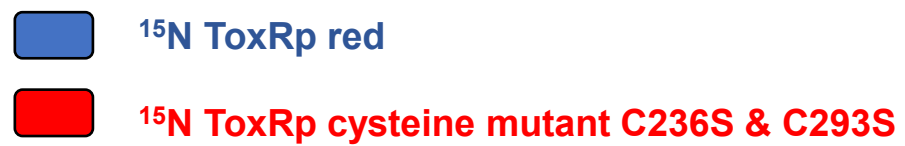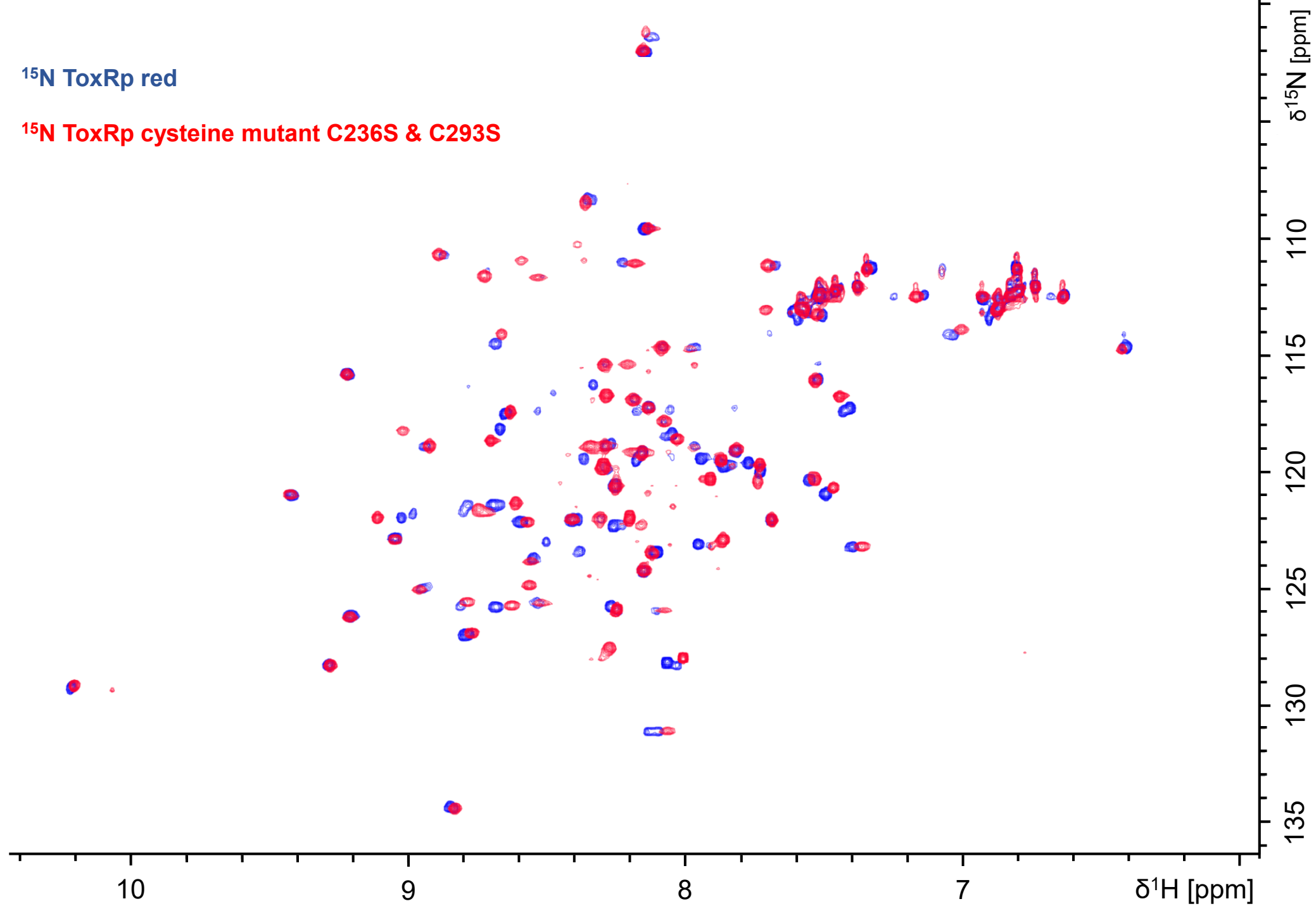

Supplement: Supplementary file 4 — Fig S4 [file MMI-115-1277-s002.pdf]
