# Supplementary material for: The periplasmic domains of Vibriocholerae ToxR and ToxS are forming a strong heterodimeric complex independent on the redox state of ToxR cysteines
Source: Mol Microbiol. 2021 Jan 25;115(6):1277–91. doi: 10.1111/mmi.14673 (PMC8359183; doi:10.1111/mmi.14673)
Supplement: Supplementary file 5 — Fig S5 [file MMI-115-1277-s004.pdf]

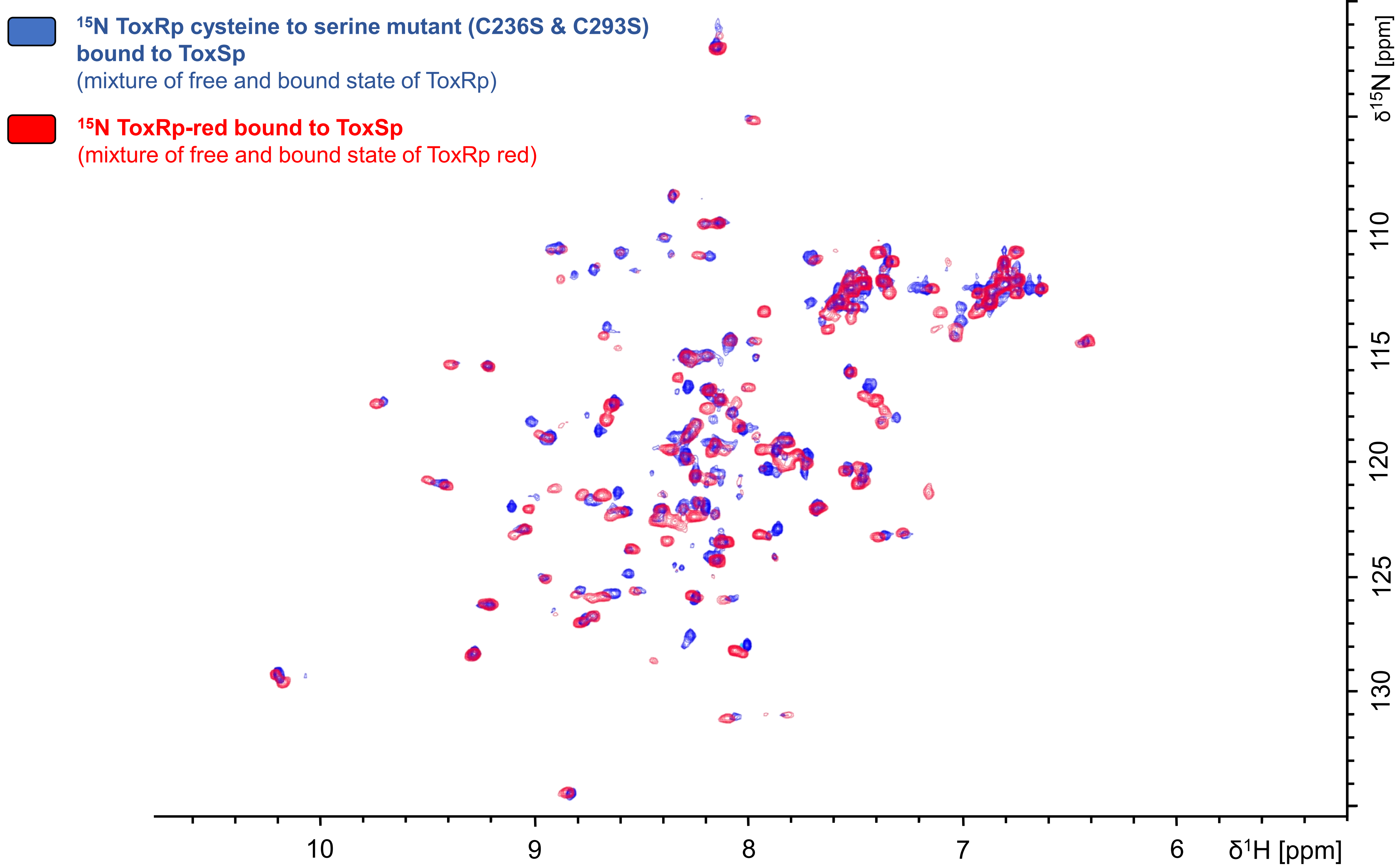
15N ToxRp cysteine to serine mutant (C236S & C293S) bound to ToxSp
(mixture of free and bound state of ToxRp)
15N ToxRp-red bound to ToxSp
(mixture of free and bound state of ToxRp red)
δ15N [ppm]
110
115
120
125
130
10
9
8
7
6
δ1H [ppm]
