# Supplementary material for: The periplasmic domains of Vibriocholerae ToxR and ToxS are forming a strong heterodimeric complex independent on the redox state of ToxR cysteines
Source: Mol Microbiol. 2021 Jan 25;115(6):1277–91. doi: 10.1111/mmi.14673 (PMC8359183; doi:10.1111/mmi.14673)
Supplement: Supplementary file 6 — Table S1 [file MMI-115-1277-s003.docx]

**Supplemental Data**

Table S1: Refinement statistics of the ToxRp-ox structure.

| ***Distance constraints*** |  |
| --- | --- |
| *Total* | 1264 (100%) |
| *Intraresidue, \|i-j\|=0* | 269 (21.3%) |
| *Sequential, \|i-j\|=1* | 354 (28.0%) |
| *medium-range, 1<\|i-j\|<5* | 199 (15.7%) |
| *long-range, \|i-j\|>=5* | 442 (35.0%) |
| ***Dihedral-angle constraints*** |  |
| *Total* | 52 |
| ***Violations*** |  |
| *RMS of distance violation / constraint* | 0.01 Å |
| *Maximum distance violation* | 0.42 Å |
| *RMS of dihedral angle violation / constraint* | 0.37 |
| *Maximum dihedral angle violation* | 3.5 |
| ***Deviations from Ideal Geometry*** |  |
| *RMS deviation for bond angles* | 0.2° |
| *RMS deviation for bond lengths* | 0.001Å |
| ***RMSD Values*** |  |
| *Backbone* | All: 6.0 Å  Ordered: 0.5 Å |
| *Heavy atoms* | All: 6.4 Å  Ordered: 0.8 Å |
| ***Ramachandran Plot*** |  |
| *Most favored regions* | 84.3% |
| *Additionally allowed regions* | 15.8% |
| *Generously allowed regions* | 0.0% |
| *Disallowed regions* | 0.0% |

Figure S1 Talos+ secondary structure prediction of monomeric ToxRp with reduced (ToxRp-red) or oxidised cysteines(ToxRp-ox). The comparison indicates that the ToxRp fold is not affected by the formation of an intramolecular disulphide bond.

Figure S2 SEC-MALS chromatogram of ToxRSp-ox. Analysis of the peak resulted in a heterodimer formation. The peak elutes after 16.19ml. The chromatogram displays light scattering (LS) at 90° angle (blue), UV absorption at 280nm (purple, no scale since arbitrary units were used) and the calculated molar mass (red).

Figure S3 Fluorescence anisotropy measurements with the ToxRSp-ox complex reveals a Kd of 11.6 Nm:

Figure S4 Overlay of ^15^N ToxRp-red (blue) and ^15^N ToxRp cysteine mutant C236S & C293S (red).

Figure S5 Overlay of ^15^N ToxRp-red bound to unlabeled ToxSp (red) and ^15^N ToxRp cysteine mutant C236S & C293S bound to unlabelled ToxSp (blue). Both spectra show a mixture of free ToxRp and ToxRp bound to ToxSp.
