# Supplementary material for: The periplasmic domains of Vibriocholerae ToxR and ToxS are forming a strong heterodimeric complex independent on the redox state of ToxR cysteines
Source: Mol Microbiol. 2021 Jan 25;115(6):1277–91. doi: 10.1111/mmi.14673 (PMC8359183; doi:10.1111/mmi.14673)

SUPPLEMENTARY INFORMATION

The periplasmic domains of *Vibrio cholerae* ToxR and ToxS are forming a strong heterodimeric complex independent on the redox state of ToxR cysteines

Nina Gubensäk[1,2], Gabriel E. Wagner-Lichtenegger[1,3], Evelyne Schrank[1], Fabio S. Falsone[1,5], Tamara Margot Ismael Berger[4], Tea Pavkov-Keller[2,4,6], Joachim Reidl[2,4,6] and Klaus Zangger[1,4,6*]

[1] Institute of Chemistry / Organic and Bioorganic Chemistry, University of Graz Heinrichstrasse 28, A-8010 Graz, Austria

[2] Institute of Molecular Biosciences, University of Graz, Humboldtstrasse 50, A-8010 Graz, Austria

[3] Diagnostic and Research Institute of Hygiene, Microbiology and Environmental Medicine, Medical University of Graz, Neue Stiftingtalstraße 6, 8010 A-Graz, Austria

[4] BioTechMed-Graz

[5] KAGes healthcare, Stiftingtalstr. 4-6, 8010 Graz

[6] Field of Excellence BioHealth – University of Graz

[*] Corresponding Author

Email: klaus.zangger@uni-graz.at

Tel: ++43 316 380-8673

Fax: ++43 316 380-9840

Table of Contents:

- **Table S1** Refinement statistics of the ToxRp-ox structure.
- **Figure S1** Talos+ secondary structure prediction of monomeric ToxRp with reduced (ToxRp-red) or oxidised cysteines (ToxRp-ox). The comparison indicates that the ToxRp fold is not affected by the formation of an intramolecular disulphide bond.
- **Figure S2** SEC-MALS chromatogram of ToxRSp-ox. Analysis of the peak resulted in a heterodimer formation. The peak elutes after 16.19ml. The chromatogram displays light scattering (LS) at 90° angle (blue), UV absorption at 280nm (purple, no scale since arbitrary units were used) and the calculated molar mass (red).
- **Figure S3** Fluorescence anisotropy measurements with the ToxRSp-ox complex reveals a Kd of 11.6 nM.
- **Figure S4** Overlay of ^{15}N ToxRp-red (blue) and ^{15}NToxRp cysteine mutant C236S & C293S (red).
- **Figure S5** Overlay of ^{15}N ToxRp-red bound to unlabeled ToxSp (red) and ^{15}N ToxRp cysteine mutant C236S & C293S bound to unlabelled ToxSp (blue). Both spectra show a mixture of free ToxRp and ToxRp bound to ToxSp.

Table S1 Refinement statistics of the ToxRp-ox structure.

Distance constraints	
Total	1264 (100%)
Intraresidue, |i-j|=0	269 (21.3%)
Sequential, |i-j|=1	354 (28.0%)
medium-range, 1<|i-j|<5	199 (15.7%)
long-range, |i-j|>=5	442 (35.0%)
Dihedral-angle constraints	
Total	52
Violations	
RMS of distance violation / constraint	0.01 Å
Maximum distance violation	0.42 Å
RMS of dihedral angle violation / constraint	0.37
Maximum dihedral angle violation	3.5
Deviations from Ideal Geometry	
RMS deviation for bond angles	0.2°
RMS deviation for bond lengths	0.001Å
RMSD Values	
Backbone	All: 6.0 Å<br>Ordered: 0.5 Å
Heavy atoms	All: 6.4 Å<br>Ordered: 0.8 Å
Ramachandran Plot	
Most favored regions	84.3%
Additionally allowed regions	15.8%
Generously allowed regions	0.0%
Disallowed regions	0.0%

Figure S1 Talos+ secondary structure prediction of monomeric ToxRp with reduced (ToxRp-red) or oxidised cysteines (ToxRp-ox). The comparison indicates that the ToxRp fold is not affected by the formation of an intramolecular disulphide bond.

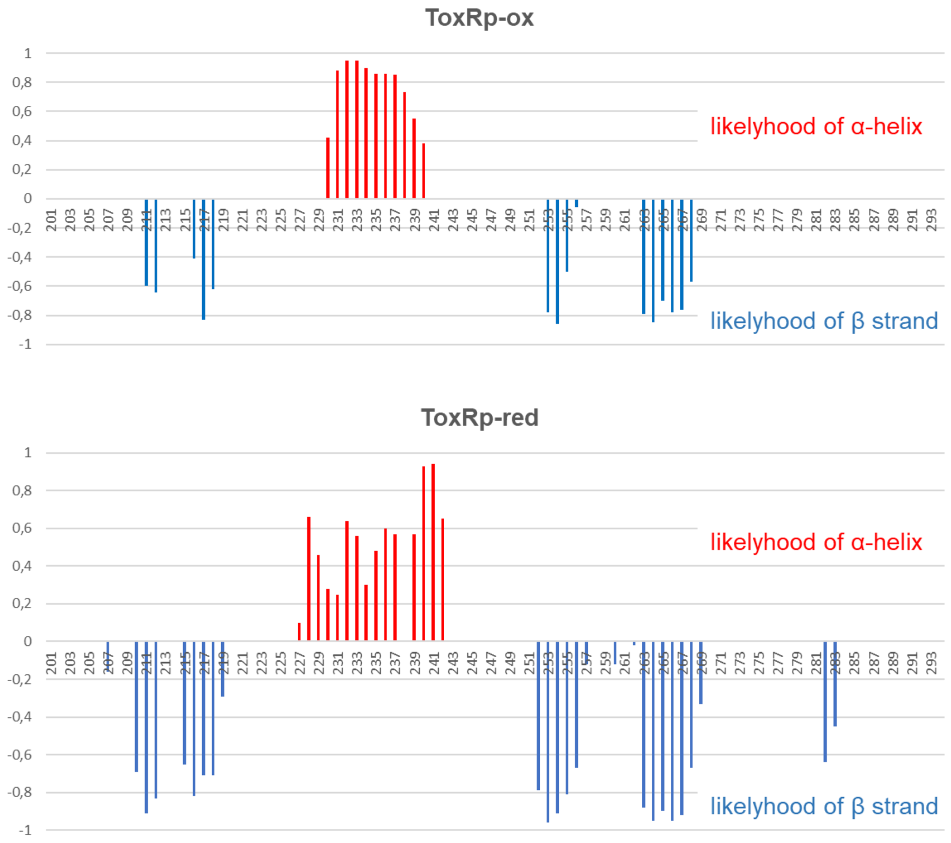

Figure S2 SEC-MALS chromatogram of ToxRSp-ox. Analysis of the peak resulted in a heterodimer formation. The peak elutes after 16.19ml. The chromatogram displays light scattering (LS) at 90° angle (blue), UV absorption at 280nm (purple, no scale since arbitrary units were used) and the calculated molar mass (red).

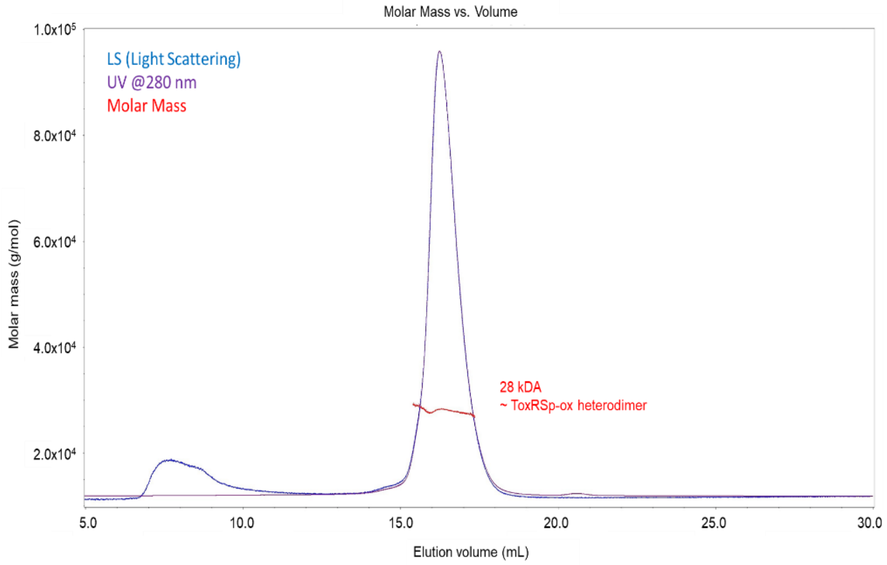

Figure S3 Fluorescence anisotropy measurements with the ToxRSp-ox complex reveals a Kd of 11.6 nM.

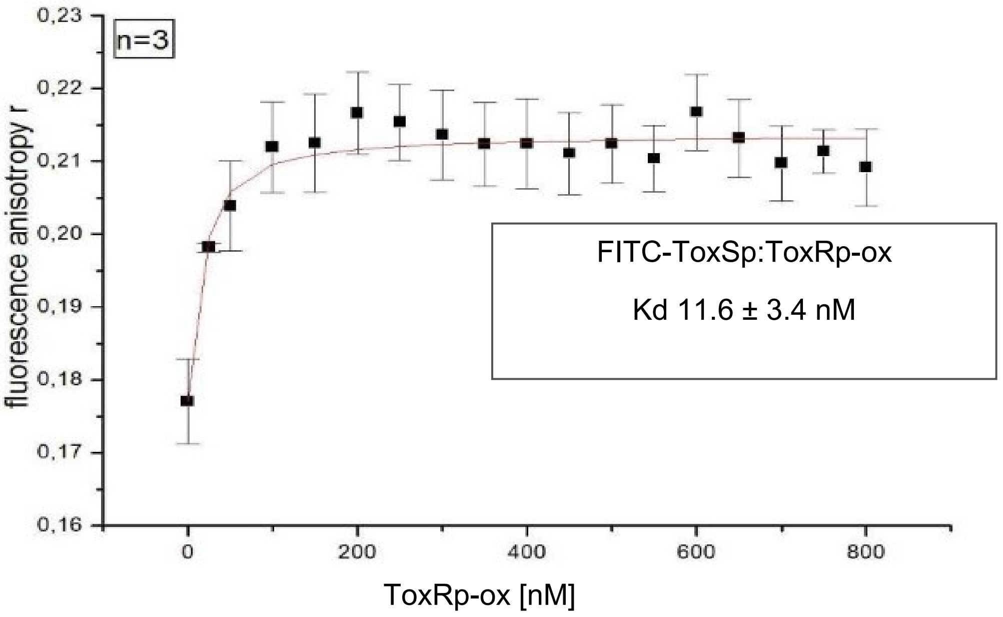

Figure S4 Overlay of ^{15}N ToxRp-red (blue) and ^{15}NToxRp cysteine mutant C236S & C293S (red).

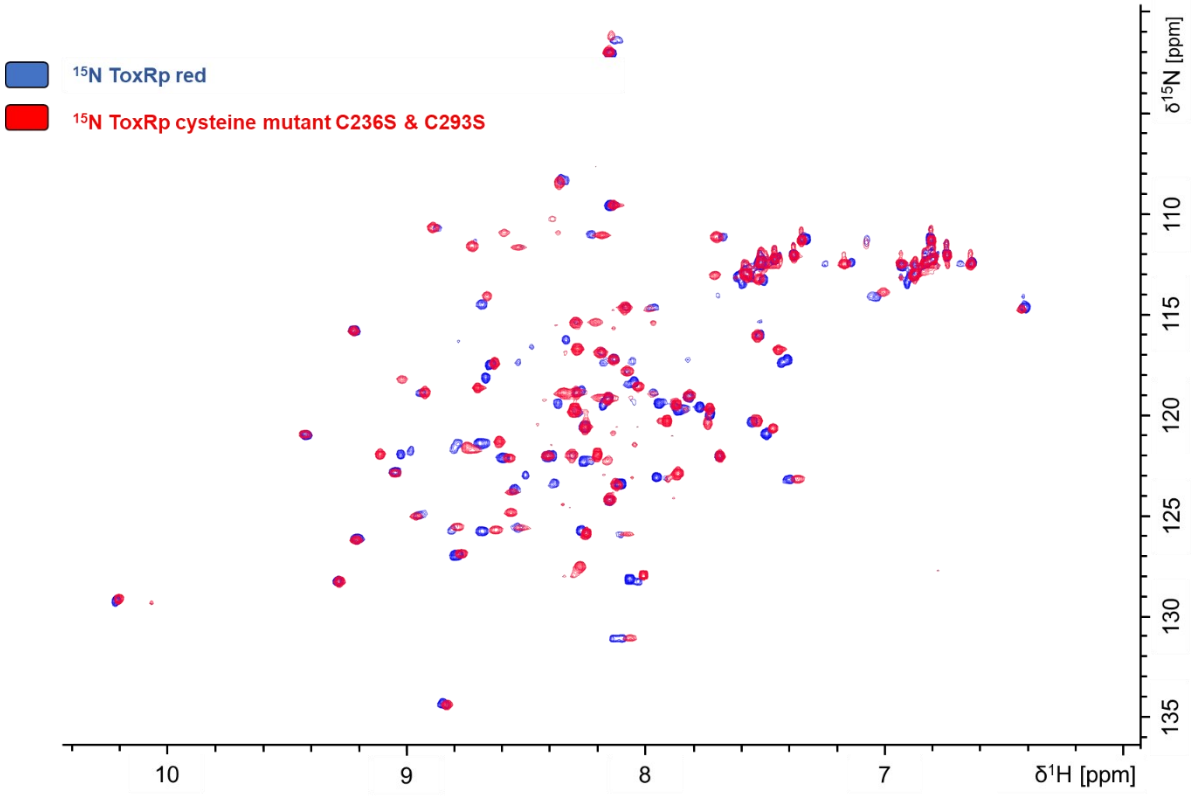

Figure S5 Overlay of ^{15}N ToxRp-red bound to unlabeled ToxSp (red) and ^{15}N ToxRp cysteine mutant C236S & C293S bound to unlabelled ToxSp (blue). Both spectra show a mixture of free ToxRp and ToxRp bound to ToxSp.

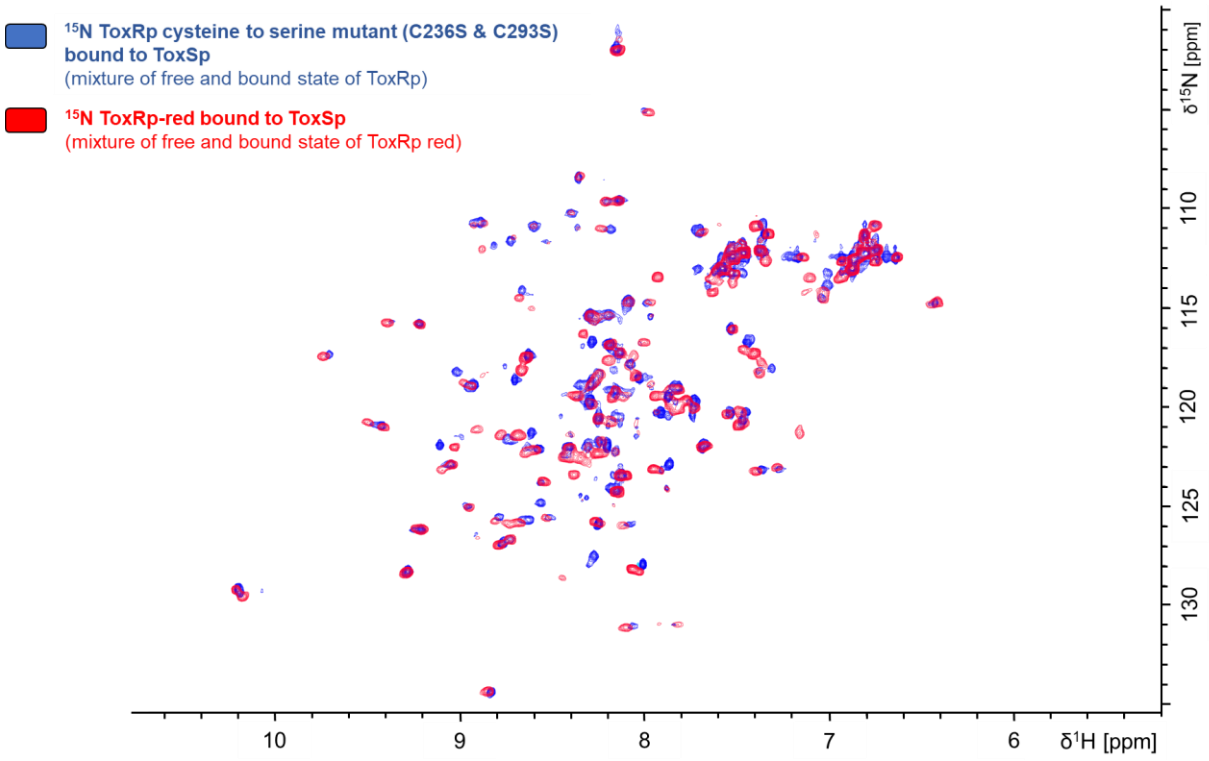

Supplement: Supplementary file 7 — Supplementary Material [file MMI-115-1277-s001.pdf]
